# Obesity-associated gut microbiome influences diet-induced metabolic and cognitive outcomes in older adults

**DOI:** 10.1080/29933935.2025.2605879

**Published:** 2025-12-25

**Authors:** Cole Patoine, Julia Sheffler, Trinity Sims, Viviana Gutierrez, Gwoncheol Park, Moses Mayonu, Bo Wang, Ravinder Nagpal

**Affiliations:** a The Gut Biome Lab, Department of Health, Nutrition, and Food Sciences, Florida State University, Tallahassee, Florida, USA; b Center for Translational Behavioral Science, Department of Behavioral Sciences and Social Medicine, Florida State University College of Medicine, Tallahassee, Florida, USA; c Department of Chemistry and Chemical Engineering, Florida Institute of Technology, Melbourne, Florida, USA; d Center of Integrative Nutrition and Food Research, Florida State University, Tallahassee, Florida, USA

**Keywords:** Aging, gut-brain axis, metabolome, microbiome, obesity

## Abstract

Obesity in older adults is a known risk factor for Alzheimer’s disease and related dementias, potentially driven by metabolic dysfunction, inflammation and gut dysbiosis. The gut-brain axis, influenced by diet and the gut microbiome, is increasingly recognized as a contributor to neurodegeneration. In this sub-analysis of a 10-week randomized dietary education intervention (NCT06121986), we examined how obesity modulates gut microbiome, metabolome, and cognitive responses in 31 adults aged 55–85, with or without mild cognitive impairment. Participants received education on either a Mediterranean Diet or a Modified Mediterranean-Ketogenic Diet. Analyses were stratified by baseline obesity (BMI ≥30 kg/m²). Individuals with obesity exhibited lower microbial alpha-diversity, higher *Bacteroides*, and lower *Akkermansia* and *Christensenellaceae_R-7_group*, along with poorer memory and executive function. Only in the obese group did fat loss correlate with improvements in episodic memory and cognitive flexibility. In contrast, increased fat mass was associated with improved memory in non-obese participants. Gains in skeletal muscle mass predicted cognitive improvement in adults aged ≥73. Changes in gut (acetate, propionate, lactate) and plasma (acetate, pyruvate, citric acid) metabolites were linked to cognitive and body composition outcomes. These exploratory findings highlight the gut-muscle-brain axis as a modifiable target to enhance cognitive health in aging populations.

## Graphical abstract

**Figure:**
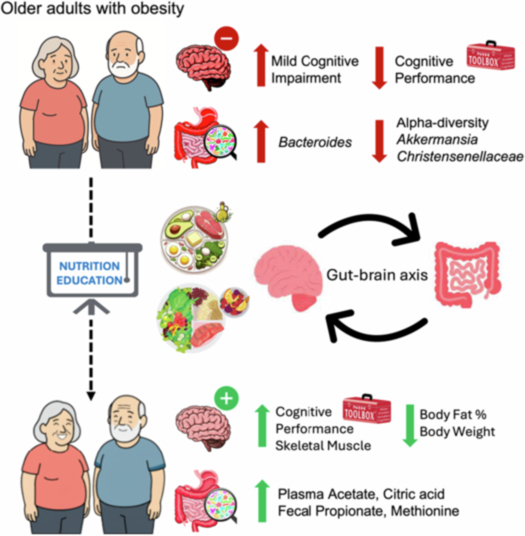

## 1 Introduction

Obesity among older adults in the U.S. has risen sharply, posing significant risks to both physical and cognitive health.^1^^,^^2^ It is a known contributor to chronic conditions like cardiovascular disease, type 2 diabetes, and metabolic syndrome,^3-5^ and growing evidence links it to increased risk for Alzheimer’s disease and related dementias (ADRDs).^6^^,^^7^ This connection is thought to stem from overlapping metabolic, inflammatory, and vascular dysfunctions that impair brain health.^8^ Chronic low-grade inflammation in obesity activates microglia and astrocytes, leading to the release of pro-inflammatory cytokines (e.g., IL-6, TNF-*α*, IL-1β), which disrupt synaptic signaling, inhibit neurogenesis, and promote accumulation of beta-amyloid and tau proteins.^8-10^ Obesity-related insulin resistance also disrupts brain insulin signaling, impairing synaptic plasticity and memory, and is increasingly recognized as a factor in Alzheimer’s pathogenesis—termed “type 3 diabetes”.^11^ Visceral fat further contributes by releasing adipokines that cross the blood-brain barrier and exacerbate neuroinflammation.^12^ Additionally, increased reactive oxygen species (ROS) promote oxidative stress, damaging neurons and impairing mitochondrial function.^13^ Obesity-induced vascular dysfunction—through hypertension, atherosclerosis, and reduced cerebral blood flow—compromises nutrient delivery and raises the risk of silent infarcts and white matter lesions.^14^ As the aging population grows, understanding obesity’s impact on brain health is an urgent public health priority.

Occurring concurrently with the development of ADRD’s, the aging process itself is associated with certain unfavorable changes in the body, including alterations in body composition, such as increased adiposity and decreased lean and skeletal mass.^15^^,^^16^ Declines in resting metabolic rate and anabolic hormones (e.g., growth hormone, testosterone, estrogen) contribute to sarcopenia, characterized by the progressive loss of muscle mass and strength.^17^ Sarcopenic obesity, characterized by increased fat and decreased muscle mass with age, has emerged as a key risk factor for older adults, and is associated with depression, mortality, impaired function, and cognitive decline.^18-21^ Even without significant weight gain, aging often leads to increased visceral fat due to adipose redistribution and reduced physical activity. This shift in body composition is linked to insulin resistance, systemic inflammation, and loss of functional independence, each a known risk factor for cognitive decline and dementia. As such, body composition measures beyond body mass index (BMI) are increasingly recognized as more accurate indicators of health status,^22^ with age-related shifts potentially heightening vulnerability to cognitive impairment.

Mild cognitive impairment (MCI) represents a transitional stage between normal cognitive aging and dementia.^23^ Individuals with MCI exhibit measurable declines in cognitive function, particularly in memory and executive functioning, that exceed typical age-related changes but do not yet significantly interfere with activities of daily living. Importantly, MCI is associated with a heightened risk of progression to Alzheimer's disease (AD), making it an essential target for early intervention strategies.^24^ Identifying modifiable risk factors like body composition, that can slow or prevent the progression from MCI to dementia is thus a major focus of current research efforts.

Diet and nutrition have also emerged as critical modifiable risk factors influencing cognitive health across the lifespan. Dietary patterns such as the Mediterranean diet (MD) that are rich in fruits, vegetables, whole grains, healthy fats (e.g., olive oil), and lean proteins, have been associated with reduced risks of obesity, metabolic dysfunction, and cognitive decline.^25-27^ This dietary pattern is characterized by high intake of polyphenols, omega-3 fatty acids, and dietary fiber, which are thought to improve vascular health, and support gut microbiota diversity, key pathways implicated in brain aging and neurodegeneration.^28-30^ Adherence to the MD has been linked to improved memory, executive functioning, and delayed progression from MCI to dementia in older adults.^31^

Beyond its metabolic effects, diet is a key modulator of the gut microbiome, a critical player in brain health via the gut-brain axis.^32^ This bidirectional system connects the gut and brain through neural, endocrine, and immune pathways, allowing gut microbes to influence brain function and vice versa.^33^ Reduced diversity and an imbalance of beneficial versus harmful bacteria (known as dysbiosis) has been linked to obesity, inflammation, and neurodegeneration.^34^^,^^35^ Specific taxa are involved: reduced *Akkermansia* and *Christensenellaceae_R-7_group* correlate with obesity and poor metabolic health, while shifts in *Bacteroides* are associated with metabolic and cognitive dysfunction.^35-40^ Aging further disrupts the microbiome, with declines in beneficial *Bifidobacterium* and *F. prausnitzii*, and increases in pro-inflammatory *Enterobacteriaceae* and *Proteobacteria*,^41-43^ driven by diet changes, medications, immune senescence, and slower gut motility.^44^ This dysbiosis may compromise gut barrier integrity, elevate systemic inflammation via endotoxins like lipopolysaccharide (LPS), and alter neuroactive metabolite production, thus linking microbial aging to cognitive decline.^32^^,^^33^ Thus, the gut microbiome may mediate the relationship between diet, metabolic health, and cognition, especially in older adults at risk for MCI and ADRDs.

The interplay between diet, obesity, the microbiome, MCI, and cognition highlights the need for integrated strategies to support cognitive resilience in later life. Obesity may worsen gut dysbiosis and metabolic dysfunction, increasing susceptibility to cognitive decline, while microbiome-targeted dietary interventions may offer protection. However, the dynamic interactions among these factors during late adulthood, a critical window for cognitive decline, remain poorly understood. Although some studies have linked metabolites and microbial signatures with BMI and weight trajectories, few have examined their relationship with cognitive changes or included diverse populations.^45-48^ The MD promotes beneficial taxa like *F. prausnitzii* and *Roseburia*, but it remains difficult to attribute these changes to specific dietary components or to connect them directly to cognitive outcomes.^49^ Moreover, obesity metrics beyond BMI, such as body composition, warrant further investigation for their relevance to cognitive health. Accumulating evidence supports a direct gut–muscle axis in which gut microbes influence skeletal muscle size and function.^50^ Mechanistic work further demonstrates that microbial metabolites regulate muscle protein turnover and anabolic resistance, linking microbiome activity to muscle quality in older adults.^51^ This emerging gut–muscle connection also interacts with the brain through shared metabolic and inflammatory pathways, suggesting an integrated brain–gut–muscle axis relevant to both cognitive and physical aging.^52^

Together, these findings highlight the need to understand how obesity-related shifts in the microbiome and microbial metabolites relate to both cognitive and physical outcomes in older adults. Thus, in the current study, we aimed to determine whether older adults with obesity exhibit greater microbiome dysbiosis and poorer cognitive performance compared to their non-obese counterparts. We also examined whether obesity status influenced changes in cognition over a 10-week dietary education intervention, including the potential protective effects of weight loss and specific microbial taxa or metabolites. Additionally, we assessed how baseline body composition related to cognitive and microbiome outcomes. We hypothesized that obesity would be associated with greater dysbiosis, reduced cognitive function, and smaller cognitive gains over time, while improvements in weight and body composition would relate to cognitive benefits and favorable microbial shifts. While many studies explore dietary patterns and cognition or the microbiome separately, few have examined how baseline obesity status may alter responsiveness to diet interventions in older adults. This sub-analysis provides novel insight into how adiposity may influence the gut-muscle-brain axis, allowing us to explore whether obesity stratification modifies cognitive or microbial outcomes independent of diet group assignment. By examining these associations, we hope to contribute to a more nuanced understanding of the modifiable biological pathways that link metabolic health, gut microbiota, and cognitive aging, ultimately informing strategies to reduce the burden of ADRDs in aging populations.

## 2 Materials and methods

### 2.1 Study design and participants

**Figure 1.**
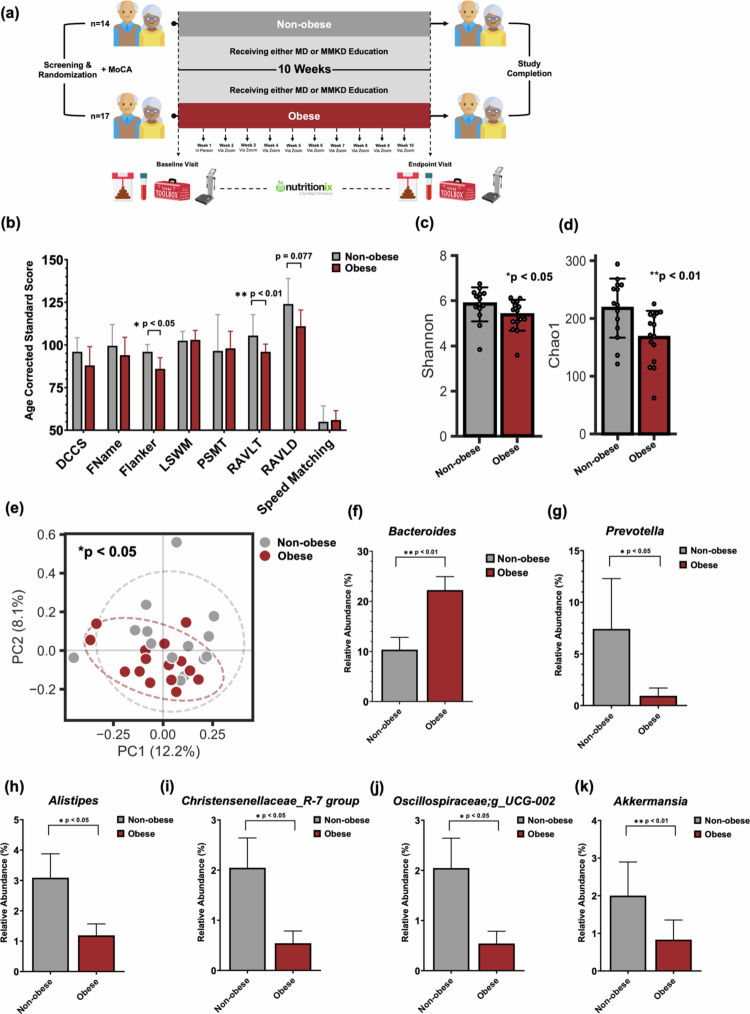
Baseline differences in cognitive performance and gut microbiota by obesity status. (a) Overview of randomized, parallel-arm study design (MoCA; Montreal Cognitive Assessment, MD; Mediterranean Diet, MMKD; Modified Mediterranean Ketogenic Diet). (b) NIH Toolbox task performance across cognitive domains shows lower age-corrected standard scores in obese (*n* = 17) group vs. non-obese (*n* = 14); between group differences tested using Mann-Whitney U tests. Data presented as median (95% CI) (c–d**)** Obese group (*n* = 15) displayed significantly reduced alpha diversity (Chao1 richness and Shannon Index) vs. non-obese (*n* = 14). (e) Beta diversity analysis (Bray-Curtis dissimilarity) revealed distinct microbial clustering by obesity status. PCoA Analysis based on Bray-Curtis Dissimilarity was used to represent Beta-diversity for each group. Significance was calculated using PERMANOVA.(f–k) Taxa-level differences in relative abundance between groups, including elevated *Bacteroides* and reduced *Akkermansia, Prevotella*, *Alistipes*, *Christensenellaceae_R-7_group,* and *Oscillospiraceae;g_UCG-002* in obese group (*n* = 15) vs. non-obese (*n* = 14); between group differences tested using Mann-Whitney U tests. Data presented as mean ± SEM.

In this randomized parallel-arm pilot clinical trial, a total of 31 participants (mean age 69.7 ± 5.6 y; 24 females, 7 males) were recruited from diverse rural communities through collaborative efforts with local organizations and institutions, including medical offices, senior centers, and nursing homes. Participants were randomly assigned to receive dietary education on either a MD or a Modified Mediterranean Ketogenic Diet (MMKD). Participants were classified as cognitively normal (CN) or having possible MCI by a trained research assistant, supervised by a licensed clinical psychologist using the Montreal Cognitive Assessment (MoCA). Achieving a MoCA score between 17 and 26 was indicative of MCI; score of >27 as CN. In this sub-group analysis of a larger, ongoing registered clinical trial (NCT06121986), we have restructured our analytic approach to investigate the impact of obesity status, rather than diet group, on gut microbiome, cognition, and related biomarkers. Study design is shown in Figure 1a. Details of participant recruitment, randomization, and retention throughout the 10-week dietary education intervention are provided in Figure S1. This study was conducted in accordance with the ethical principles outlined in the Declaration of Helsinki, the Belmont Report, and the guidelines of the International Committee of Medical Journal Editors (ICMJE) on the protection of research participants. The protocol was reviewed and approved by the Institutional Review Boards of Florida State University (IRB protocol #STUDY00003781). Written informed consent was obtained from all participants prior to their enrollment in the study. Participants were informed of the study objectives, procedures, potential risks, and their right to withdraw at any time without penalty. All data were anonymized to ensure confidentiality. The study was conducted at the Center for Translational Behavioral Science, housed within Department of Behavioral Sciences and Social Medicine in the Florida State University College of Medicine.

### 2.2 Screening and eligibility

Participants were primarily recruited through collaborative efforts with local community organizations, as well as word-of-mouth and flyers. A standardized 30-minute telephone screening was completed with all potential participants, which included questions related to the eligibility criteria listed below: (1) participants aged between 55 and 85 y; (2) a MoCA score ranging from 17−26, indicative of possible MCI, or a score of 27 or higher for CN individuals; (3) expressed interest in the program; (4) stability in medical conditions; (5) stability in medication regimens as evaluated by the study clinician; and (6) the capacity to complete the required assessments throughout the duration of the study. The exclusion criteria included (1) a MoCA score of 16 or lower; (2) any diagnosis of a neurodegenerative disorder; (3) a history of head injury accompanied by loss of consciousness or presence of a brain lesion; (4) major psychiatric disorders, including psychosis, bipolar disorder, or issues related to substance abuse; (5) sensory impairments that may prevent the participant from engaging in the intervention; (6) serious medical risks as determined by the study clinician; (7) use of prescribed warfarin, insulin, antibiotics, or immunosuppressant medications; (8) utilization of anticonvulsant medications or other substances potentially affecting the central nervous system; (9) dietary restrictions, such as allergies to nuts or fish; (10) adherence to restrictive or specialized dietary regiments; (11) significant digestive disorders; 12) previous surgical interventions that could complicate dietary interventions; and (13) an inability to communicate effectively in English. Upon completion of the initial screening call, individuals identified as eligible participants were contacted to engage in the informed consent process.

### 2.3 Intervention

Participants engaged in a 10-week program of weekly 1-hour group sessions via HIPAA-compliant Zoom, led by a Registered Dietitian and trained research assistant using Motivational Interviewing (MI) and Cognitive Behavioral Therapy (CBT) techniques. The initial 90-minute session was conducted in person and included a psychoeducational video, discussion, and nutrition education. The topics covered included: Nutrition fundamentals, MD or MMKD principles, strategies for addressing factors related to eating, physical activity, rest, and behavioral strategies for healthy lifestyle changes. All participants received a workbook containing meal plans, grocery lists, handouts, and tracking tools for food, and macronutrient intake, as well as access to an online forum designed to facilitate recipe sharing and provide additional social support beyond live group meetings, with pertinent information on accessing this forum disseminated through email correspondence from the laboratory. Overall, education in both groups emphasized high-quality, nutrient-dense foods and promoted fiber intake as key components to fostering adherence and long-term behavior change. These diets were not designed to illicit weight loss, rather promote whole-food dietary improvements and standardize nutrient quality across participants. Given this, rather than evaluate diet-specific mechanisms, the current analysis focused on obesity status and microbiome–cognition relationships, wherein outcomes were examined collectively across diet groups. Prior to the intervention, participants were asked to download a food tracking application (Nutritionix). All participants were required to document their dietary intake using the app over three days each week (two weekdays and one weekend) during the 10-week intervention period. Participants who had trouble with the app were provided 3-d food logs in their workbooks to use instead. Although participants were encouraged to log dietary intake on 3 d/week, inclusion in final dietary analyzes required at least one usable day per week. Participants without at least one usable day of dietary data each week were excluded from analysis. Dietary intake data were ultimately available for 22 participants.

### 2.4 Body composition analysis

Body weight and measures of body composition were measured at baseline and the 10-week endpoint. Body weight was measured in kilograms, and body composition was assessed using a multi-frequency bioelectrical impedance analysis (BIA) device (Mediana i55 Premium Body Composition Analyzer, Mediana Co., Ltd., South Korea), which is validated for research applications. The analyzer provided estimates of fat mass, lean body mass, skeletal muscle mass, and total body water. Measurements were performed according to manufacturer guidelines, with participants standing barefoot on the analyzer platform and holding the hand electrodes. Prior to assessment, participants were instructed to avoid strenuous exercise, food, and fluid intake for at least 2 hours to minimize variability. We employed BIA due to its portability, lower cost, and feasibility for repeated measures in community-dwelling older adults. We acknowledge that dual-energy X-ray absorptiometry (DEXA) provides more precise estimates of fat distribution and lean mass but was not feasible within the scope of this pilot study. Participants were asked to stand on the BIA machine barefoot, grasping the hand electrodes and ensuring proper alignment of their feet with the foot electrodes. The participant held this position for approximately 1 to 1.5 minutes while the machine computed the relevant data, including fat mass (kg) or expressed as a percentage (body fat percentage), muscle mass (kg), skeletal mass (kg), total body water (L), visceral fat, and subcutaneous fat. Body mass index was calculated as body weight (kg)/height in meters squared. Obesity was defined as having a BMI greater than or equal to 30.0 kg/m^2^. Percent-based metrics (e.g., % skeletal muscle mass) were not available from the device output and could were not calculated post hoc.

### 2.5 Cognitive evaluation

Participants completed a series of selected cognitive assessments from the NIH Toolbox Cognition Battery, administered on iPads by a trained research assistant. Selected, standardized tests were administered at baseline and at 10-week follow-up. Tests were selected to evaluate multiple domains of cognitive functioning using validated, age-normed tools suitable for individuals aged 3–85. The battery included eight tests: 1. The Dimensional Change Card Sort Test (DCCS), which assessed cognitive flexibility and attention by having participants match images based on shifting dimensions. 2. The Face Name Associative Memory Exam (FName) Delay tested associative memory by requiring participants to recall 12 face-name pairs after a five-minute delay, measuring memory retention and retrieval. 3. The Flanker Inhibitory Control and Attention Task (Flanker) measured inhibitory control and attention across 20 to 40 trials, where participants must focus on a central target while ignoring peripheral distractions. 4. The List Sorting Working Memory Test (LSWM) evaluated working memory by having participants recall and sequence images of foods and animals by size. 5. The Picture Sequence Memory Test (PSMT) assessed episodic memory through the recall of picture sequences ranging from 6 to 18 images, awarding credit for each correctly ordered pair. 6. The Rey Auditory Verbal Learning Test (RAVLT) and 7. Rey Auditory Verbal Learning Delay (RAVLD) measured immediate memory and verbal learning by asking participants to recall a list of 15 unrelated words across three trials, followed by a five-minute delayed recall. 8. The Speed Matching Test evaluated processing speed by requiring participants to identify the image that differs from the others, with higher scores indicating faster cognitive processing. All cognitive assessments were scored using age-corrected standard scores, where a score of 100 reflects the national average. Scores of 115 or 85 represent one standard deviation above or below the mean, respectively.

### 2.6 Fecal collection and microbiome analysis

Gut microbiome profiles were measured as per our previously described methods.^53-58^ Briefly, participants were provided with a fecal sample collection kit, along with detailed instructions for sample collection at baseline and the 10-week endpoint. Participants collected freshly voided fecal samples (~1-2 g) as per the kit instructions after each baseline and post visit. Samples were frozen at -80 °C immediately after being delivered to our Gut Biome Lab or picked up by lab research personnel within 8 hours of defecation, while being kept cold from the time of collection to delivery using pre-frozen ice-packs. High-quality genomic DNA was extracted from 200 mg of fecal material using the PowerFecal Pro DNA kit (Qiagen Inc., Valencia, CA), following the manufacturer’s instructions and quantified using a nanodrop spectrophotometer. To target the bacterial community, the V4 hypervariable region of the microbial 16S ribosomal RNA gene was amplified using barcoded universal primers 515F and 806 R, in accordance with the benchmark Earth Microbiome Project standard protocol (http://www.earthmicrobiome.org). The resulting amplicons were purified using AMPure® magnetic purification beads (Agencourt) and quantified using a Qubit 4 fluorometer (Invitrogen). Equimolar concentrations of each library were then pooled, and the final amplicon library was subjected to paired-end sequencing (2 × 250 bp) on an Illumina MiSeq platform using the MiSeq Reagent Kit v3 (Illumina Inc., San Diego, United States). The QIIME2 (Quantitative Insights into Microbial Ecology) ver. 2-2024.10 software package (www.qiime2.org) was used for microbiome analysis. Raw sequencing reads were demultiplexed and quality-filtered using the q2-demux plugin, followed by trimming and denoising with DADA2.^59^ All resulting amplicon sequence variants (ASVs) were aligned using MAFFT.^60^ Taxonomic classification of ASVs was performed using the sklearn classifier and a pre-trained naïve Bayes taxonomy model, trained on the 99% SILVA ver. 138 (data trained: May 30, 2024; Sklearn version 1.4.2).^61^ ASVs were then collapsed to the genus level, and all subsequent analyzes were performed at the genus level.

### 2.7 Metabolomic analysis

Fecal and plasma metabolomes were measured using the global untargeted approach, as per our previously described methods.^56-58^^,^^62^ Briefly, for plasma metabolome, fasting blood samples were drawn at baseline and the 10-week endpoint visit by a trained phlebotomist using a lithium heparin tube. This tube was inverted 6-8 times post-collection and subsequently spun in a cold centrifuge for 15 minutes at 2800 rpm. Plasma was then pipetted into 0.5 ml microtubes and immediately frozen at –80 °C, and stored without additional freeze–thaw cycles until subsequent metabolomic analysis. Metabolomic profiles were measured using a global untargeted approach via a high-throughput Nuclear Magnetic Resonance (NMR) system. Stool samples were processed according to a previously established protocol,^63^ with minor modifications. Briefly, samples were extracted by vortexing with deionized water for five minutes. The resulting extracts were mixed with a phosphate buffer (pH 7.4) in D₂O to yield a final solution containing 10% D₂O, 0.1 M phosphate buffer, and 0.1 mM trimethylsilyl propionate (TSP). After centrifugation, the supernatants were transferred into 5 mm NMR tubes and analyzed using a Bruker Ascend 400 MHz high-resolution NMR system (Bruker BioSpin, Germany). For all samples, a 1D NOESY experiment (noesygppr1d) with water suppression was performed, using 64 scans. NMR data were processed in TopSpin 4.06 (Bruker BioSpin) with phasing and referencing to TSP. Spectral analysis was carried out using Amix 4.0 (Bruker BioSpin), where automated binning was applied to reduce peak overlap and splitting, as previously described.^64^ Metabolites were quantified using Chenomx 8.6 (Chenomx Inc.), and total intensity normalization was performed prior to downstream data analysis.

### 2.8 Statistical analysis

Statistical analyzes were performed using the SPSS Statistics 29.0 Software Package (SPSS Inc.). All data were initially assessed for normality using the Shapiro-Wilk test. For variables that violated normality assumptions (*p* < 0.05), non-parametric tests were employed. Wilcoxon matched-pairs signed-rank tests (two-tailed) were used to assess within-group changes from baseline to endpoint, while Mann–Whitney U tests (two-tailed) were used to compare differences between the obese and non-obese groups at either baseline or endpoint. Relative abundance comparisons of microbial features at the phylum, order, class, family, and genus levels were conducted using non-parametric tests to detect differentially abundant taxa between the obese and non-obese groups. Alpha-diversity metrics, including Chao1 (richness) and Shannon Index (richness and evenness), were calculated based on ASVs. In metabolomic analysis, data were log2-transformed to express fold changes between pre- and post-intervention levels, where the log2 fold change was calculated as log2 (Post/Pre). A positive value indicated increased metabolite levels post-intervention, while a negative value indicated decreases, and a value of 0 indicated no change. The significance of changes in metabolite concentrations was assessed using Wilcoxon signed-rank tests, with Benjamini-Hochberg false discovery rate (FDR) correction applied for multiple comparisons. To assess relationships between continuous variables, Spearman’s rank-order correlation was used, given that the data did not meet the assumptions for parametric correlation techniques. Analyzes compared participants with and without obesity and assessed within-group baseline to post-intervention changes; therefore, Mann Whitney U tests were used for between-group comparisons and Wilcoxon signed-rank tests for paired comparisons. Repeated measures ANOVA or Friedman tests were not applied, as the study did not evaluate diet-group effects or time-by-group interactions and included only two time points. Linear regression was employed to examine the strength and direction of significant associations between continuous outcome variables, while logistic regression was used for categorical outcomes. Additionally, Analysis of Covariance (ANCOVA) was applied to evaluate group differences and control for potential confounding variables, such as age and baseline cognitive performance. Potential covariates were evaluated using a priori based on their established relevance to cognitive performance, body composition, and metabolic outcomes in aging research. Candidate covariates included age, sex, years of education, baseline cognitive scores, diet group assignment, and MCI status. Each covariate was first tested individually using univariate screening: Spearman correlations were used for continuous covariates and Mann Whitney U tests for categorical variables. Covariates showing associations with any outcome at *p* < 0.10 were retained for further evaluation. Multicollinearity among candidate covariates was assessed, and variables demonstrating high intercorrelation (r > 0.70) were not entered simultaneously to avoid redundancy. Final ANCOVA and regression models included only covariates that were both statistically relevant in univariate. Additionally, to evaluate the longitudinal and group-specific effects of the dietary intervention on metabolite concentrations, we conducted three-way mixed ANOVAs with Diet (MD vs MMKD) and Obesity status (obese vs non-obese) as between-subject factors and Timepoint (baseline vs endpoint) as a repeated within-subject factor. This model generated main effects (Diet, Obesity, Timepoint) and interaction terms (Diet × Timepoint, Obesity × Timepoint, Diet × Obesity, and Diet × Obesity × Timepoint). These analyzes allowed us to assess whether and how metabolite trajectories differed over time, varied by obesity status, or were modified by diet. Dietary intake data was calculated by taking the average reported intake of all nutrients over the course of the entire 10-week intervention. Analyzes were conducted on available data; no data imputation was performed. Participants with missing outcome or covariate data were excluded from the relevant analyzes, and sample sizes are reported accordingly Statistical significance was set at *α* = 0.05 for all analyzes.

## 3 Results

### 3.1 Participant characteristics and cognitive status

At baseline, demographic characteristics were similar between participants with obesity and those without obesity (Table 1). However, obesity status demonstrated a moderate association with baseline cognitive status. While not reaching statistical significance, there was a trend indicating that participants with obesity were more likely to be classified as MCI than their non-obese counterparts. Using Fisher’s exact test, the association approached significance (*p* = 0.076). Specifically, 71% (12/17) of obese participants met criteria for MCI, whereas only 36% (5/14) non-obese participants (36%) were classified as such.

**Table 1.** Baseline characteristics of non-obese and obese participants (*n* = 31)^1^

Variable	Non-obese (*n* = 14)	Obese (*n* = 17)	*p*-value
Age (years)	71.5 (68.5, 76.5)	67.0 (65.0, 72.0)	0.084
Sex, *n* (%) Male Female	2 (14%) 12 (86%)	5 (29%) 12 (71%)	0.412
Education, *n* (%) Did Not Complete High School High School Diploma Some College Undergraduate Degree Master’s Degree Doctoral Degree	0 (0%) 1 (7%) 1 (7%) 4 (29%) 5 (36%) 3 (21%)	1 (6%) 2 (12%) 3 (18%) 7 (41%) 4 (23%) 0 (0%)	0.40
Race, *n* (%) White Black Other	7 (50%) 5 (36%) 2 (14%)	8 (47%) 9 (53%) 0 (0)	0.229
Cognitive Status, *n* (%) Cognitively Normal Mild Cognitive Impairment	9 (64%) 5 (36%)	5 (29%) 12 (71%)	0.076
Body Mass Index (kg/m^2^)	23.9 (22.9, 25.7)	36.9 (33.2, 39.1)	<0.01**
BMI Category Underweight Normal Weight Overweight Obese (Class 1) Obese (Class 2) Obese (Class 3)	1 (7%) 8 (57%) 5 (36%) 0 (0%) 0 (0%) 0 (0%)	0 (0%) 0 (0%) 0 (0%) 7 (41%) 7 (41%) 3 (18%)	<0.01

**p < 0.01, Mann-Whitney U Test or Fisher’s Exact, (Non-obese vs. Obese).

1 Data presented as median (IQR) for continuous variables and n (%) for categorical variables.

### 3.2 Baseline clinical characteristics

As anticipated, participants with obesity exhibited significantly higher values across a range of anthropometric and body composition metrics compared to non-obese participants (Table 2). Specifically, participants with obesity had significantly greater body weight (*p* < 0.01) and body fat percentage (*p* < 0.01), as well as elevated levels of visceral fat (*p* < 0.01) and increased subcutaneous fat (*p* < 0.001), indicating both central and peripheral adiposity. In addition, those with obesity demonstrated significantly higher total body water (*p* < 0.05), muscle mass (*p* < 0.05), and skeletal muscle mass (*p* < 0.05), likely reflecting their greater overall body size and weight-bearing demands. These findings are consistent with established physiological differences between obese and non-obese individuals and confirm the classification criteria used in this study. In terms of cognitive performance, participants with obesity scored lower than participants without obesity across five out of eight tasks of the NIH Toolbox Cognition Battery (DCCS, FName, Flanker, RAVLT, and RAVLD, all *p* < 0.05), which assessed a broad spectrum of neurocognitive domains including cognitive flexibility/attention, associative memory, inhibitory control, and immediate memory and verbal learning, respectively (Figure 1b, Table 3). Among these, two tasks revealed statistically significant between-group differences: Flanker (*p* < 0.05), which assesses selective attention and inhibitory control, and RAVLT (*p* < 0.01), a measure of verbal learning and memory. These results suggest that obesity may be associated with diminished cognitive functioning, particularly in executive function and memory domains.

**Table 2.** Baseline and post-intervention body-composition metrics by obesity status (*n* = 31)^1^

Variable	Group	Baseline	Endpoint^2^	Within-group *p*-value	Between-group *p*-value (Baseline)	Between-group *p*-value (Endpoint)^2^
Weight (kg)	Non-Obese Obese	66.35 (61.2, 66.4) 104.6 (89.9, 119.9)	62.3 (60.1, 75.6) 101.9 (90.8, 120)	<0.01** <0.05*	<0.01**	<0.01**
Body Fat (%)	Non-Obese Obese	35.2 (27.5, 39.0) 46.45 (37.6, 50.6)	35.3 (29.6, 38.1) 43.7 (34.0, 47.4)	0.142 0.091	<0.01**	<0.05*
Muscle Mass (kg)	Non-Obese Obese	40.0 (36.4, 54.3) 51.8 (43.6, 64.3)	38.7 (36.35, 47.6) 58.7 (47.6, 66.55)	0.480 0.929	<0.05*	<0.01**
Skeletal Muscle (kg)	Non-Obese Obese	21.9 (19.35, 31.9) 30.45 (24.2, 39.2)	20.9 (19.2, 27.2) 35.0 (27.6, 40.95)	0.288 0.783	<0.05*	<0.01**
Protein Content (kg)	Non-Obese Obese	8.25 (7.55, 11.2) 10.5 (8.45, 12.1)	8.2 (7.65, 9.95) 12.2 (8.25, 13.8)	0.99 0.213	0.110	<0.05*
Mineral Content (kg)	Non-Obese Obese	3.13 (2.9, 4.0) 3.85 (3.0, 4.5)	3.11 (2.83, 3.55) 4.22 (3.19, 5.12)	<0.05* 0.610	0.145	<0.05*
Total Body Water (L)	Non-Obese Obese	31.25 (28.6, 42.3) 40.2 (37.7, 51.4)	30.2 (28.3, 37.0) 43.6 (37.85, 50.3)	0.366 0.721	<0.05*	<0.01**
Visceral Fat Index	Non-Obese Obese	1.0 (1.0, 2.0) 3.0 (3.0, 3.0)	1.0 (1.0, 2.0) 3.0 (3.0, 3.0)	0.564 0.317	<0.01**	<0.01**
Subcutaneous Fat (cm^2^)	Non-Obese Obese	185.6 (153.2, 202.9) 419.9 (323.8, 491.4)	177.7 (160.4, 213.3) 385.7 (276.4, 472.2)	<0.05* <0.01**	<0.01**	<0.01**

Within-group significance: Paired Wilcoxon Signed Ranks Test, *p < 0.05, **p < 0.01.

Between-group significance: Mann-Whitney U Test, (Non-obese vs. Obese), *p < 0.05, **p < 0.01.

1 Data presented as median (IQR) for continuous variables and n (%) for categorical variable.

2 Data only available for n=13 of obese group and n=13 of non-obese group.

**Table 3.** Baseline and post-intervention cognitive performance by obesity status (*n* = 31)^1^

Variable	Group	Baseline	Endpoint^2^	Within-group *p*-value	Between-group *p*-value (Baseline)	Between-group *p*-value (Endpoint)^2^
DCCS	Non-Obese Obese	96.9 (86.25, 104.25) 88.0 (79.5, 99.0)	101.0 (88.5, 107.0) 90.0 (83.5, 103.0)	0.151 0.125	0.138	0.243
FName	Non-Obese Obese	99.5 (87.25, 112.0) 94.0 (89.9, 104.5)	113.5 (102.3, 122.8) 104.0 (99.5, 115.0)	<0.05* <0.01**	0.570	0.247
Flanker	Non-Obese Obese	96.0 (88.0, 100.25) 86.0 (81.0, 92.5)	99.0 (93.0, 105.5) 85.0 (82.0, 99.0)	0.421 0.875	<0.05*	<0.05*
LSWM	Non-Obese Obese	102.5 (95.75, 108.0) 103.0 (91.0, 108.5)	105.0 (97.5, 114.5) 102.0 (91.5, 109.5)	0.237 0.380	0.984	0.545
PSMT	Non-Obese Obese	96.5 (89.5, 117.75) 98.0 (91.5, 108.0)	96.0 (78.5, 107.0) 100.0 (92.5, 109.5)	<0.05* 0.133	0.953	0.287
RAVLT	Non-Obese Obese	105.5 (99.0, 117.75) 100.5 (89.9, 96.0)	110.0 (105.5, 115.0) 103.0 (95.0, 108.5)	0.593 < 0.05*	<0.01**	<0.05*
RAVLD	Non-Obese Obese	124.0 (107.8, 139.0) 111.0 (104.0, 120.5)	121.0 (110.0, 137.5) 113.0 (101.5, 125.0)	0.456 0.241	0.077	0.223
Speed Matching	Non-Obese Obese	55.0 (48.5, 64.25) 56.0 (44.5, 61.5)	58.0 (48.5, 71.5) 56.0 (54.0, 65.75)	0.133 0.099	0.830	0.894

Within-group significance: Paired Wilcoxon Signed Ranks Test, *p < 0.05, **p < 0.01. Between-group significance: Mann-Whitney U Test, (Non-obese vs. Obese), *p < 0.05, **p < 0.01. All variables presented as age corrected standard scores.

1 Data presented as median (IQR).

2 Data only available for *n* = 13 of obese group and *n* = 13 of non-obese group.

### 3.3 Obesity-associated alterations in microbiome and metabolome

Obesity status was significantly associated with alterations in gut microbiome. Participants with obesity exhibited reduced alpha-diversity, as indicated by significantly lower Chao1 richness indices (*p* < 0.05) and Shannon diversity (*p* < 0.05) suggesting a less diverse and potentially less resilient gut (Figure 1c,d). In addition, beta-diversity analysis using Bray-Curtis dissimilarity revealed distinct clustering of microbial communities between obese and non-obese cohorts, reflecting significant compositional differences in microbial taxa at the community level (Figure 1e). Taxonomic analysis at the genus level further revealed specific microbial shifts associated with obesity. Relative abundance (%) of genus *Bacteroides* was significantly higher in obese participants compared to non-obese counterparts (*p* < 0.01) (Figure 1f), while several beneficial or health-associated taxa were significantly reduced in the obese group. These included *Prevotella* (*p* = 0.03), *Alistipes* (*p* < 0.05), *Christensenellaceae_R-7 group* (*p* < 0.05), *Oscillospiraceae;g_UCG-002* (*p* < 0.05), and *Akkermansia* (*p* < 0.01), (Figure 1g,k). Many of these taxa have previously been implicated in maintaining gut barrier integrity, modulating host metabolism, suggesting that their depletion may contribute to the metabolic and cognitive consequences observed in obesity. When participants were stratified by cognitive status, further patterns emerged. Among individuals with MCI, obese-MCI participants had significantly higher levels of *Bacteroides* (*p* < 0.05) and significantly lower levels of *Akkermansia* (*p* < 0.05) compared to non-obese-MCI participants. In contrast, among CN participants, most taxa differences were attenuated; only the *Christensenellaceae_R-7 group* remained significantly reduced in the obese-CN group (*p* < 0.05), suggesting this taxon may be particularly sensitive to obesity status independent of cognitive classification. At baseline, fecal and plasma metabolite concentrations did not differ significantly between obese and non-obese participants. However, several metabolites demonstrated trends toward differential abundance that may warrant further investigation in larger cohorts. Notably, plasma glutamine levels were higher in the obese group compared to the non-obese group, approaching statistical significance (*p* = 0.085). Conversely, plasma pyruvate levels tended to be lower in the obese group (*p* = 0.072).

### 3.4 Dietary adherence and intake

Throughout the 10-week intervention, there were no significant between-group differences in total nutrient intake, indicating similar levels of dietary adherence among participants with and without obesity (Table 4). However, dietary pattern analyzes revealed that participants with obesity reported higher intake of eggs and lower intake of nuts compared to the non-obese group, suggesting minor but potentially meaningful differences in food choices that may reflect dietary preferences or habitual patterns. Mediterranean Diet Adherence Screener (MEDAS) scores were comparable between groups (obese: mean = 5.93; non-obese: mean = 6.00), suggesting similar overall adherence to a mediterranean style diet. A more detailed comparison of nutrient and food group intake by obesity status is provided in Supplementary Table 1 (S1)**.**

**Table 4.** Dietary intake profiles of obese and non-obese participants during the 10-week intervention (*n* = 22)^1^

Variable	Non-obese (*n* = 11)	Obese (*n* = 11)	*p*-value
Kcals	1381 (1158, 1804)	1501 (943, 1843)	0.847
Protein (g)	59.9 (52.3, 78.4)	64.6 (50.0, 74.0)	0.949
Total Fat (g)	74.69 (53.28, 79.7)	72.51 (45.45, 80.09)	0.652
Saturated Fat (g)	18.49 (15.02, 20.94)	20.16 (10.79, 22.07)	0.699
Monounsaturated Fatty Acids (g)	24.43 (19.83, 29.58)	26.31 (18.09, 30.46)	0.949
Polyunsaturated Fatty Acids (g)	21.15 (16.87, 26.76)	18.02 (13.39, 21.68)	0.217
Carbohydrate (g)	124.3 (110.26, 136.08)	156.72 (97.34, 182.55)	0.606
Caffeine (mg)	27.9 (2.56, 103.6)	41.35 (4.3, 158.3)	0.562
Total Sugars (mg)	58.94 (40.65, 68.59)	65.54 (42.53, 74.05)	0.562
Fiber (g)	15.05 (11.82, 23.57)	13.55 (11.09, 19.85)	0.30
Calcium (mg)	573.0 (397.73, 791.0)	497.87 (439.0, 635.69)	0.797
Iron (ng)	9.45 (8.0, 11.8)	9.80 (7.65, 12.65)	0.652
Magnesium (mg)	308.64 (187.3, 393.1)	204.24 (177.4, 319.97)	0.243
Potassium (mg)	2110.0 (1433.0, 2652.9)	1840.9 (1500.4, 2662.6)	0.797
Sodium (mg)	1758.5 (1508.5, 2005.5)	2399.2 (1517.3, 3101.3)	0.116
Zinc (mg)	8.1 (6.2, 11.24)	7.05 (5.32, 9.24)	0.401
Copper (mg)	1.39 (0.73, 1.67)	0.88 (0.70, 1.14)	0.076
Eggs (oz eq.)	0.18 (0.0, 0.73)	0.83 (0.54, 1.45)	<0.05*
Nuts (oz eq.)	3.2 (1.71, 3.84)	0.74 (0.40, 1.9)	<0.05*
MEDAS Score (Endpoint)	6.00 (4.50, 8.00)	5.50 (5.00, 7.00)	0.981

Mann-Whitney U Test, (Non-obese vs. Obese), *p < 0.05, **p < 0.01.

1 Data presented as Median (IQR).

### 3.5 Post-intervention differences in body composition

**Figure 2.**
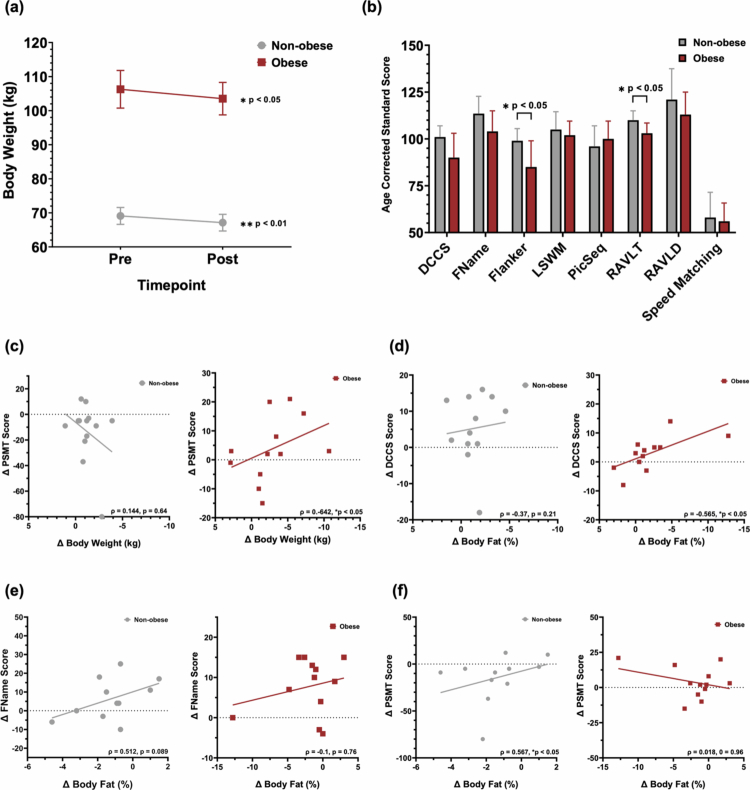
Post-intervention weight loss and cognitive gains stratified by obesity status. (a) Weight loss by group over 10-week intervention, with greater absolute loss in the obese group (*n* = 13) vs. non-obese (*n* = 13); within-group changes tested with Wilcoxon signed-rank tests; between-group comparisons tested with Mann–Whitney U tests. Data presented as mean ± SEM. (b) Post intervention Flanker and RAVLT scores remain significantly lower in the obese group (*n* = 13) vs. non-obese (*n* = 13); between-group comparisons tested with Mann–Whitney U tests. Data presented as median (95% CI). (c) Negative correlation between weight loss and PSMT improvement in obese group compared to non-obese group. (d) Fat mass reduction is associated with gains in executive function (DCCS) in obese group. (e,f) In non-obese participants, fat gain is paradoxically associated with improved PSMT and FName performance. (c–f) Correlations between body composition changes and cognitive performance assessed using Spearman rank-order correlations.

At the end of the 10-week intervention, the obese group continued to exhibit significantly higher values across a range of anthropometric and body composition measures compared to their non-obese counterparts. These included body weight (*p* < 0.01), muscle mass (*p* < 0.01), body fat mass (*p* < 0.05), total body water (*p* < 0.01), visceral fat (*p* < 0.01), skeletal muscle mass (*p* = 0.01), and subcutaneous fat (*p* < 0.01). In addition, post-intervention assessments revealed new between-group differences in protein content (*p* < 0.05) and mineral content (*p* < 0.05), with higher values in the obese group. These findings are consistent with baseline differences and reflect the persistence of obesity-related body composition characteristics following the intervention period. Both groups experienced significant within-group weight loss over the course of the study. Participants with obesity lost an average of 5.95 kg (pre vs. post, *p* < 0.05), whereas non-obese participants lost an average of 1.22 kg (pre vs. post, *p* < 0.01) (Figure 2a). A new variable was created to represent weight change over the 10- week intervention (post weight (kg)—pre weight (kg)). Using non-parametric Mann Whitney U test, grouping by obesity status, it was found that the obese group numerically lost more weight compared to the non-obese group, although this result was not statistically significant (*p* = 0.057). Despite this difference in absolute weight change, within-group comparisons revealed no statistically significant pre vs. post changes in specific body composition metrics for either group, including fat mass and skeletal muscle mass. This suggests that while modest weight loss occurred, it was not accompanied by significant shifts in any specific compartmental body composition over the 10-week period. It is worthy to note that weight loss was not a goal or hypothesized outcome of the study intervention in either group.

### 3.6 Domain-specific cognitive gains across obesity groups

Depicted in Table 3, cognitive performance improved over the intervention period in both groups, with within-group gains observed in specific cognitive domains. The Wilcoxon signed-rank test was used to assess pre- to post-intervention changes in test scores for the obese and non-obese groups separately. Among obese participants, significant improvements were observed on the FName (*p* < 0.01) and the RAVLT (*p* < 0.05), indicating enhancements in associative and verbal memory, respectively. A trend toward improved processing speed was noted on the Speed Matching task (*p* = 0.099). In the non-obese group, significant gains were found on the FName task (*p* < 0.05) and the PSMT (*p* < 0.05), suggesting improvements in episodic memory and associative learning. Between-group comparisons revealed that the Flanker (*p* < 0.05) and RAVLT (*p* < 0.05) scores remained significantly lower in the obese group compared to the non-obese group, indicating that obesity may have a persistent impact on these cognitive measures (Figure 2b). Notably, both tests showed lower scores at baseline in the obese group, suggesting that obesity could have an ongoing detrimental effect on attention, inhibition, and memory performance, which are assessed by the Flanker and RAVLT tasks, respectively. To further explore this, a MANCOVA was conducted to examine whether obesity status was associated with post-intervention cognitive performance (Flanker and RAVLT scores), controlling for education level, MCI status, and baseline test performance. After adjusting for covariates, obesity status did not have a significant effect on Flanker performance, *F*(1, 20) = 0.090, *p* = .768, η² = .004, or RAVLT performance, *F*(1, 20) = 0.043, *p* = .838, η² = .002. Baseline performance was a significant predictor for both Flanker (*F*(1, 20) = 20.595, *p* < .001, η² = .507) and RAVLT (*F*(1, 20) = 6.593, *p* = .018, η² = .248) outcomes. No other covariates were significant.

### 3.7 Body composition changes predict cognitive gains in older adults with obesity

Among obese participants, body composition changes were significantly associated with improvements in specific cognitive domains. Consistent with the Spearman correlation results, which indicated that greater weight loss was associated with larger improvements in PSMT performance (negative *ρ* reflects scoring direction) (*ρ* = –0.642, *p* < 0.05; Figure 2c), the ANCOVA analysis further supported this association. After adjusting for age, education, and baseline PSMT scores, weight loss remained a significant predictor of improved episodic memory in the obese group (F(1,7) = 8.776, *p* < 0.05), but not in the non-obese group. These findings suggest that larger reductions in body weight were linked to greater memory gains specifically among participants with obesity. Reductions in body fat percentage were significantly associated with improved performance on the DCCS task, which assesses cognitive flexibility and executive function (*ρ* = –0.676, *p* < 0.05) (Figure 2d). To expand upon these correlational results, a linear regression analysis was conducted to explore the relationship between changes in body fat mass and performance on the DCCS task separately for obese and non-obese participants. In obese participants, reductions in body fat mass were significantly associated with improvements in DCCS performance (*β* = -0.660, *p* < 0.05). Specifically, for each one-unit decrease in body fat mass, DCCS scores were predicted to increase by 0.951 points. This negative relationship suggests that reductions in body fat mass were linked to enhanced cognitive flexibility in this group. These findings suggest that improvements in body composition may be linked to enhanced cognitive outcomes in metabolically compromised individuals. In contrast, no such associations were observed in non-obese participants. Paradoxically, in this group, increased body fat was positively correlated with improved PSMT scores (*ρ* = 0.567, *p* < 0.05) (Figure 2e), and a non-significant trend was noted with FName performance (*ρ* = 0.512, *p* = 0.089) (Figure 2f). Age-stratified analyzes revealed differential effects of body composition on total cognitive change. Among older participants (aged 73–80), increases in skeletal muscle mass significantly predicted total cognitive change (B = 19.544, SE = 3.872, t = 5.047, *p* < 0.01) (Figure S2), supporting the hypothesis that preservation or gain of lean mass in advanced age may have neurocognitive benefits. In contrast, this relationship was not observed in the younger age stratum (aged 58–72), where skeletal muscle change was unrelated to total cognitive change (*p* = 0.851). These findings suggest that the link between muscle and memory may become more pronounced with aging, possibly reflecting the compounding impact of sarcopenia and metabolic frailty on brain health. Although, these patterns represent correlational relationships only and do not imply that changes in adiposity directly caused changes in cognitive performance.

### 3.8 Nutrition education intervention alters microbiome and metabolome distinctly in non-obese and obese individuals

**Figure 3.**
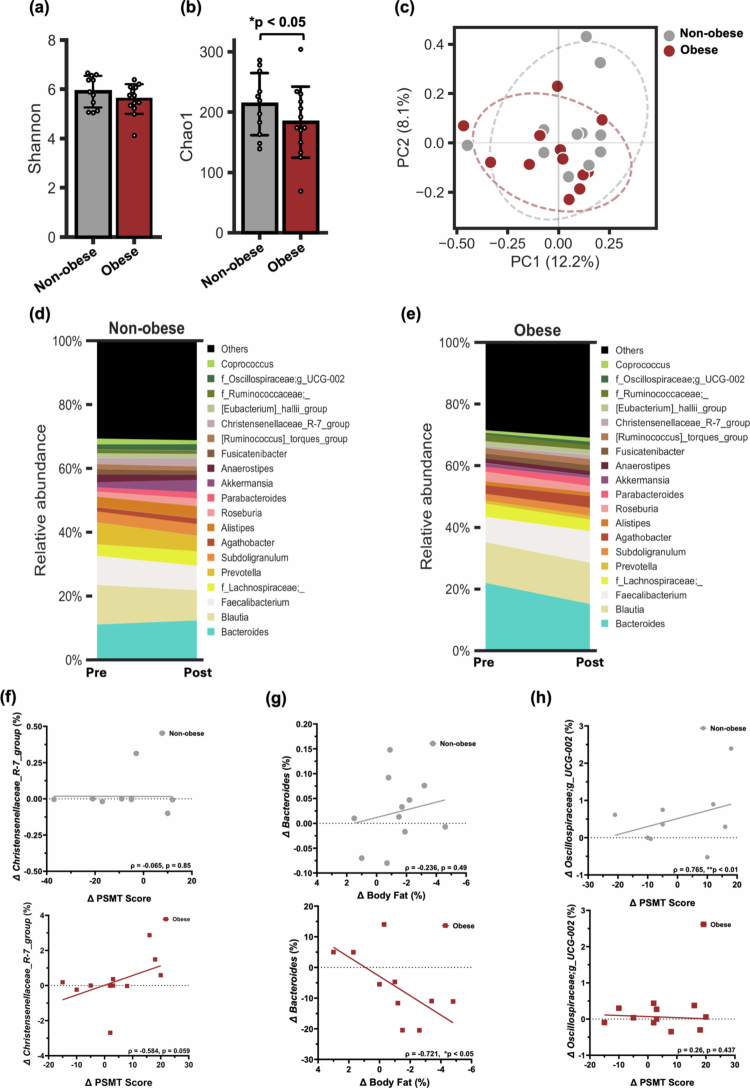
Diet-induced shifts in gut microbiota and their associations with cognitive and body composition outcomes. (a–c) post-intervention changes in Shannon diversity, Chao1 richness, and beta diversity; microbial richness remains lower in the obese group (*n* = 12) vs. non-obese (*n* = 11). (d–e) Genus-level shifts pre-to-post intervention stratified by obesity status. (f) *Christensenellaceae_R-7_group* increase shows trend toward improved PSMT scores in participants with obesity. (g) Reduction in *Bacteroides* correlates with fat loss in participants with obesity. (h) Increase in *Oscillospiraceae;g_UCG-002* linked to better episodic memory in non-obese.(f–h) Correlations between body composition changes and cognitive performance assessed using Spearman rank-order correlations.

After the 10-week nutrition education intervention, differences in gut microbiome between participants with and without obesity diminished for Shannon diversity (Figure 3a). In contrast, microbial richness measured by the Chao1 index remained significantly lower in the obese group (*p* < 0.05), indicating a persistent deficit in species richness (Figure 3b). Differences in beta-diversity based on Bray–Curtis dissimilarity also diminished, showing reduced separation between groups after the intervention (Figure 3c). This suggests that obesity may exert lasting effects on specific aspects of gut microbial composition, particularly species richness, despite dietary modulation. At baseline, the Firmicute/Bacteroidota (F/B) phylum ratio for the obese group was 2.18 (F/B: 65.1%/29.9%), while the non-obese group had a slightly higher ratio of 2.94 (F/B: 69.9%/23.8%). After a 10-week dietary education intervention, the obese group showed a notable increase in the F/B ratio, which rose to 3.52 (F/B: 73.6%/20.9%). This increase reflects a reduction in the relative abundance of Bacteroidota and a corresponding increase in Firmicutes. Conversely, the non-obese group showed a slight decrease in the ratio, which dropped to 2.75 (F/B: 66.4%/24.1%), suggesting a modest shift toward greater Bacteroidota relative to Firmicutes.

To assess whether metabolite trajectories differed by diet and obesity status over time, we conducted three-way mixed ANOVAs with diet (MD vs MMKD) and obesity status (obese vs non-obese) as between-subject factors and Timepoint (baseline vs endpoint) as the repeated within-subject factor. Full ANOVA outputs for all metabolites are provided in Supplementary Table 2 (S2). Only two metabolites, fecal lactate and fecal choline, showed a significant diet × obesity × timepoint interaction, indicating that baseline-to-endpoint changes in these metabolites depended jointly on diet assignment and obesity status. No other metabolites demonstrated this three-way interaction.

### 3.9 Microbial shifts predict body composition and cognitive outcomes, varying by obesity status

Changes in the gut microbiome at the genus level in participants with and without obesity are shown in Figure 3d and 3e, respectively (phylum level changes are depicted in Figure S3 and S4). Associations between gut microbial taxa and both body composition and cognitive function were also observed and varied by obesity status. Among individuals with obesity, increases in *Christensenellaceae_R-7_group* abundance showed a trend with improved PSMT scores (*ρ* = 0.584, *p* = 0.059) (Figure 3f). Reductions in *Bacteroides* were associated with both lower carbohydrate intake (*ρ* = -0.733, *p* < 0.05) and greater loss of body fat (*ρ* = -0.721, *p* < 0.05) (Figure 3g). Furthermore, a binary logistic regression was conducted to examine whether the relative abundance of *Bacteroides* could predict obesity status while controlling for MCI status. The model was statistically significant for *Bacteroides* abundance, indicating that it was a significant predictor of obesity (B = –10.301, SE = 4.598, Wald = 5.019, *p* < 0.05), suggesting that higher *Bacteroides* abundance was associated with substantially reduced odds of being classified as obese. MCI status was not a significant predictor (B = 1.115, SE = 0.925, Wald = 1.456, *p* = .228), though the odds ratio (OR = 3.051, 95% CI [0.498, 18.680]) indicated a possible trend toward increased obesity risk among participants with MCI. In the non-obese group, increased energy intake was inversely associated with levels of *Akkermansia* (*ρ* = –0.709, *p* < 0.05) and *Christensenellaceae_R-7_group* (*ρ* = –0.624, *p* = 0.054). Notably, increased abundance of *Oscillospiraceae;g_UCG-002* was strongly correlated with improved PSMT performance (*ρ* = 0.765, *p* < 0.01) (Figure 3h), highlighting a potential role for this taxon in modulating cognitive function. To explore whether changes in key gut microbial taxa could predict cognitive improvements, multiple linear regression models were constructed within the obese subgroup. A model incorporating changes in *Bacteroides*, *Alistipes*, *Akkermansia*, and *Christensenellaceae_R-7_group* as predictors of episodic memory (as measured by the PSMT) identified *Christensenellaceae_R-7_group* as a marginal, but not significant contributor (B = 620.239, t = 2.093, *p* = 0.081).

### 3.10 Obesity-dependent metabolomic associations with cognitive performance

**Figure 4.**
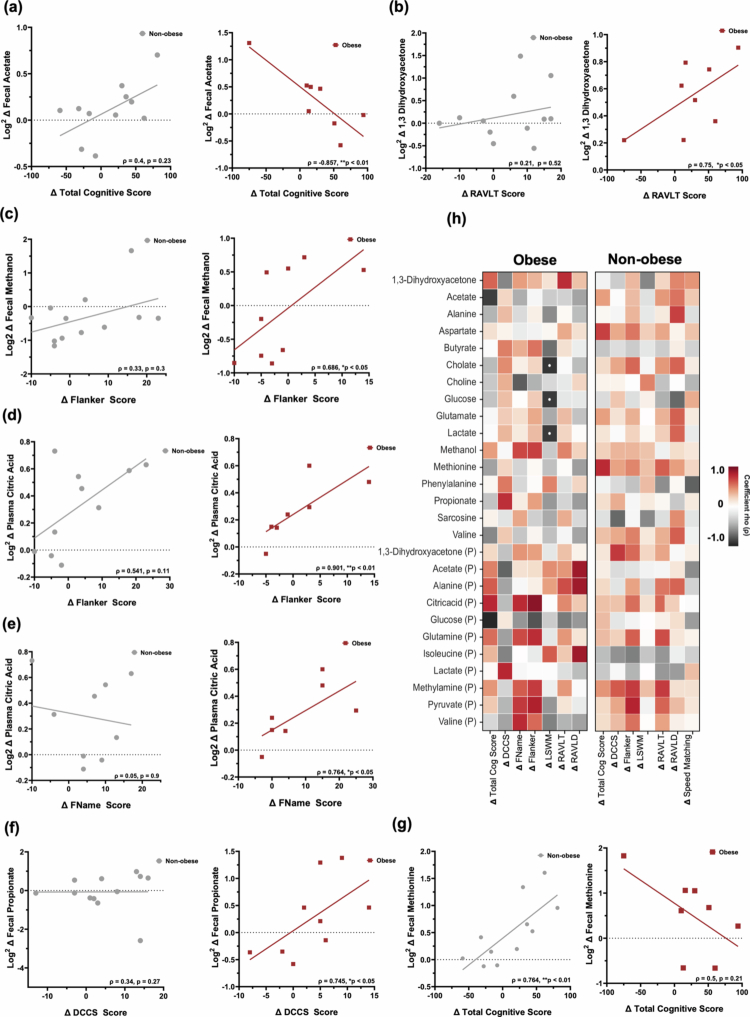
Metabolite-cognitive associations by obesity status.(a) Negative correlation between fecal acetate and global cognitive change in obese group. (b) Fecal 1,3-dihydroxyacetone positively associated with RAVLT performance in obese group. (c) Fecal methanol linked to improved Flanker scores in obese group.(d–e) Plasma citric acid associated with Flanker and FName performance in participants with obesity.(f) Fecal propionate correlates with DCCS performance in participants with obesity. (g) In non-obese group, fecal methionine linked to total cognitive improvement. (h) Heatmap depicting the correlational patterns between Log2 fold changes in fecal and plasma metabolites versus change total cognitive scores and domain specific scores. (Spearman rank-order correlation, FDR-corrected *p*-values reported; * *p* < 0.05).

Distinct relationships between metabolic, microbial, and cognitive changes also emerged when stratifying participants by obesity status. To evaluate overall cognitive change, we computed a composite “total cognitive change” score for each participant by summing age-corrected standard scores from all eight NIH Toolbox cognitive tests at baseline and again at week 10. The difference between the post-intervention and baseline totals (Week 10 total—Baseline total) was used as the primary indicator of global cognitive change, with positive values indicating improvement. In the obese group overall, cognitive improvement was significantly and inversely correlated with fecal acetate (*ρ* = –0.857, *p* < 0.01) (Figure 4a). Beyond global cognitive change, specific metabolites showed domain-specific associations. For example, fecal 1,3-dihydroxyacetone was positively associated with performance on the Rey Auditory Verbal Learning Test (RAVLT; *ρ* = 0.75, *p* < 0.05) (Figure 4b), implicating it in verbal memory.

Similarly, fecal methanol was associated with improved scores on both the Flanker task (*ρ* = 0.686, *p* < 0.05) (Figure 4c). Plasma citric acid was another metabolite positively associated with Flanker (*ρ* = 0.901, *p* < 0.01) and FName performance (*ρ* = 0.764, *p* < 0.05), linking systemic energy metabolism with cognitive function (Figure 4d,e). Plasma pyruvate also showed strong correlations to improved performance on those two tests (*p* < 0.05). Fecal propionate was also tied to better cognitive flexibility as measured by the DCCS test (*ρ* = 0.745, *p* < 0.05) (Figure 4f). Finally, plasma acetate, alanine, and isoleucine were all tied to improved RAVLD scores (*p* < 0.05). In the non-obese group, a distinct pattern emerged, with improved total cognitive change being positively associated with fecal methionine (*ρ* = 0.764, *p* < 0.01) (Figure 4g), with a similar association between plasma pyruvate and Flanker scores seen again, as previously found with the obese group (*ρ* = 0.765, *p* < 0.05). A comprehensive heatmap depicting the correlational patterns between Log2 fold changes in fecal and plasma metabolites versus change total cognitive scores and domain specific scores is shown by Figure 4h. For completeness, we also generated an unstratified heatmap depicting the correlational patterns between Log2 fold changes in fecal and plasma metabolites and all outcome domains across the entire cohort (Figure S5). This figure is provided to complement the obesity-stratified analyzes presented in the main results.

### 3.11 Body composition changes correlate with distinct metabolomic signatures

**Figure 5.**
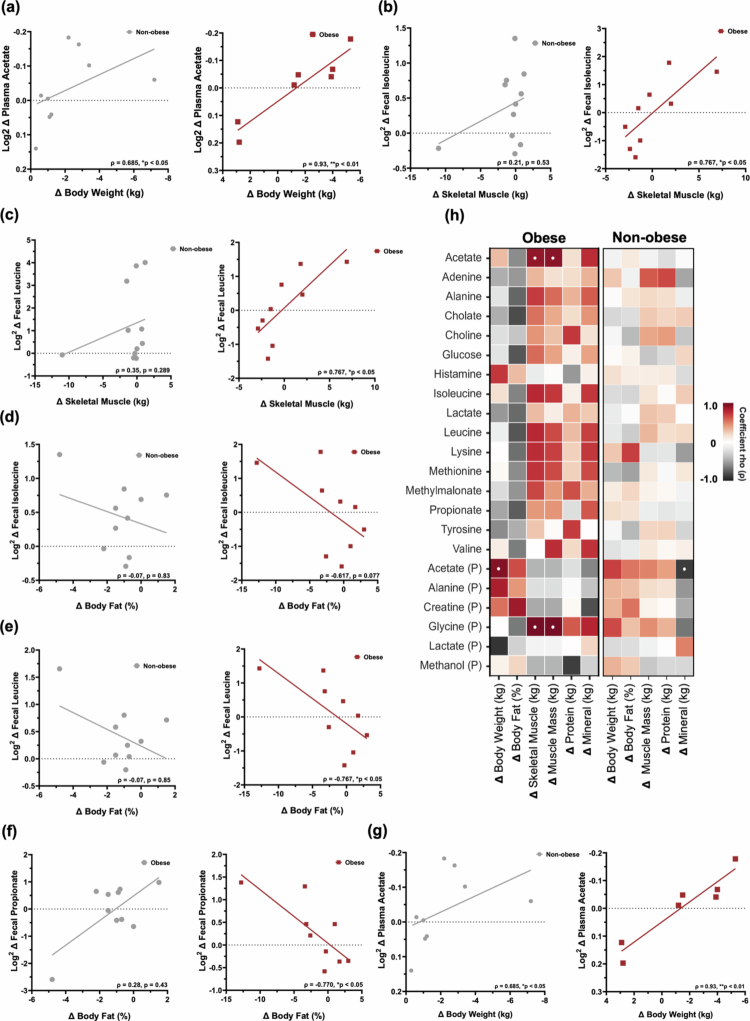
Metabolite signatures and changes in body composition. (a) Plasma acetate correlates with weight loss in both groups. (b–e) Increases in fecal BCAAs (e.g., leucine, isoleucine) and other amino acids correlate with muscle gain and reduced body fat in participants with obesity. (f) Fecal propionate negatively associated with body fat in participants with obesity. (g) Plasma acetate correlates with weight loss in both groups.(h) Heatmap depicting the correlational patterns between Log2 fold changes in fecal and plasma metabolites versus change in body composition metrics. (Spearman rank-order correlation, FDR-corrected *p*-values reported; *p < 0.05).

In obese participants, several metabolites demonstrated significant associations with changes in body composition following the intervention (Figure 5). Plasma acetate emerged as a strong predictor of weight loss (B = 27.439, SE = 4.833, 95% CI [6.643, 48.236], t = 5.677, *p* < 0.05) (Figure 5a). The standardized beta (*β* = 1.049) indicates a strong positive association between plasma acetate and weight loss. Additionally, reductions in plasma lactate (*ρ* = –0.893, *p* < 0.01), and increases in plasma alanine (*ρ* = 0.821, *p* < 0.05), and fecal histamine (*ρ* = 0.717, *p* < 0.05) were each significantly correlated with weight loss, suggesting a multifaceted metabolic response involving both systemic and gut-derived compounds. A suite of amino acids—including fecal alanine, isoleucine, leucine, lysine, valine and methionine—also positively correlated with increases in skeletal muscle mass (*ρ* = 0.700 to 0.767) and inversely correlated with changes in body fat percentage (*ρ* = –0.617 to –0.770) (Figure 5b,e). Notably, leucine, isoleucine and lysine further exhibited positive associations with mineral content, suggesting broader implications for musculoskeletal integrity. Fecal propionate demonstrated an inverse association with body fat (*ρ* = –0.770, *p* < 0.05) (Figure 5f). In contrast, within the non-obese group, no significant relationships were observed between amino acid changes and body composition besides one opposite association between elevated fecal lysine and increased body fat percentage, which contrasts to obese participants. However, weight loss in this group was linked to a reduction in plasma acetate (*ρ* = 0.685, *p* < 0.05), mirroring findings in the obese cohort and reinforcing acetate’s relevance to metabolic shifts across phenotypes (Figure 5g). A heatmap depicting the correlational patterns between Log2 fold changes in fecal and plasma metabolites versus change in body composition metrics is shown by Figure 5h.

### 3.12 Microbiome-metabolome co-regulation arrays suggest coordinated functional networks

**Figure 6.**
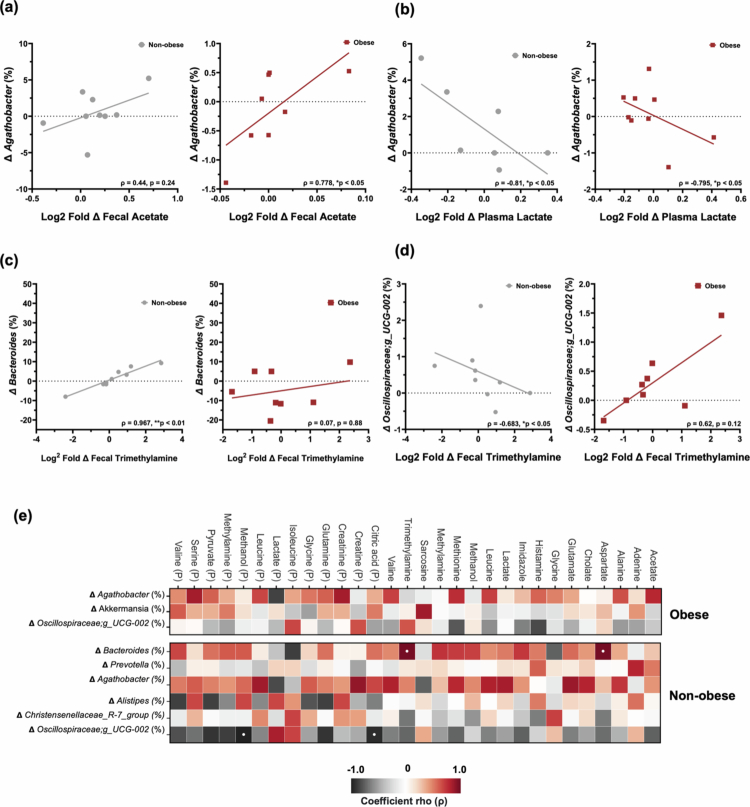
Microbiome–metabolite co-associations suggest functional networks. (a–b) In obese group, *Agathobacter* correlates positively with fecal acetate and negatively with plasma lactate. (c–d) In non-obese group, trimethylamine is positively associated with *Bacteroides* and negatively with *Oscillospiraceae;g_UCG-002*.(e) Heatmap illustrating associations between metabolite shifts and microbial taxa across groups (Spearman rank-order correlation, FDR-corrected *p*-values reported; * *p* < 0.05).

Several key microbial-metabolite relationships emerged, revealing coordinated shifts in the gut ecosystem associated with functional changes in host metabolism and cognition. Among obese participants, the genus *Agathobacter* consistently displayed positive correlations with fecal acetate (*ρ* = 0.778, *p* < 0.05) (Figure 6a), and inverse relationships with plasma lactate (*ρ* = −0.795, *p* < 0.05) (Figure 6b), suggesting that this SCFA-producing genus may serve as a central hub in microbiome-metabolome interactions and associated metabolite production and signaling. In non-obese participants, trimethylamine (TMA) showed opposing associations with the bacterial genera *Bacteroides* and *Oscillospiraceae;g_UCG-002*, being strongly positively correlated with *Bacteroides* abundance (*ρ* = 0.967, *p* < 0.001) (Figure 6c) and negatively correlated with *Oscillospiraceae;g_UCG-002* (*ρ* = –0.683, *p* < 0.05) (Figure 6d). A heatmap depicting the correlational patterns between Log2 fold changes in fecal and plasma metabolites versus change in taxa (relative abundance %) is shown by Figure 6e.

## 4 Discussion

This study aimed to explore the relationships between obesity status, cognitive performance, gut microbiota, and metabolites in older adults. The findings contribute to a growing body of evidence suggesting that obesity may have complex effects on cognitive function, gut microbiome composition, and metabolic pathways. Obesity is a complex condition that not only contributes to metabolic dysfunction but also impacts various aspects of cognitive health, particularly in older adults. Growing evidence suggests that obesity may have direct and indirect effects on brain function through various pathways, including the gut microbiome. In recent years, research has highlighted the role of the gut microbiome as a key player in obesity-related health outcomes, linking it to metabolic diseases and even cognitive decline.^65^

### 4.1 Obesity-associated cognitive deficits and gut dysbiosis in older adults

Our data showed that participants with obesity demonstrated lower performance on multiple cognitive tasks, with significant impairments observed in executive function (Flanker) and verbal memory (RAVLD). These results align with existing literature linking obesity to diminished cognitive control and memory performance, particularly in aging populations.^66^^,^^67^ Interestingly, when participants with MCI were excluded, the observed cognitive deficits in the obese group were no longer significant. This attenuation suggests that the impact of obesity on cognitive performance may be mediated or amplified by MCI status, indicating that obese individuals may be more vulnerable to cognitive decline and that obesity could be a contributing risk factor in the progression from normal cognition to MCI. Future longitudinal studies should investigate whether obesity accelerates cognitive decline over time, and whether early interventions in obese but cognitively normal individuals can prevent or delay MCI onset. Obesity was associated with reduced gut microbial alpha-diversity and distinct community structures, consistent with prior findings.^68^ These changes reflect a less diverse and potentially less resilient microbiome, which may contribute to impaired gut barrier integrity and increased systemic inflammation, both of which are implicated in the pathogenesis of cognitive decline, although not directly measured in this study. Specifically, the obese group showed reduced abundance of several taxa associated with metabolic and neurological health, including *Prevotella*, *Alistipes*, *Christensenellaceae R-7 group*, *Oscillospiraceae UCG-002*, and *Akkermansia*. A 2022 meta-analysis found strikingly similar results to our study, showing that obese individuals have significantly altered microbiomes, with a reduced abundance of 23 genera, such as *Christensenellaceae_R-7_group*, *Alistipes*, and *Akkermansia.*^69^*Akkermansia muciniphila* is a mucin-degrading bacterium known for its role in maintaining gut barrier integrity and regulating host metabolism. It has emerged as a promising biomarker and therapeutic target in obesity and metabolic syndrome.^70^ By consuming mucin, *Akkermansia* stimulates mucus layer renewal, reinforcing gut barrier function and preventing endotoxemia and influences glucose and lipid metabolism through pathways involving GLP-1, adiponectin, and AMPK activation.^70^ Although once considered a next-generation probiotic, recent literature suggests its impact on host health depends on host genetics, strain-specific properties, and interactions with other microbes.^71^^,^^72^ Reductions in *Prevotella, Oscillospiraceae (g_UCG-002), and Alistipes* suggest diminished microbial functionality relevant to metabolic balance and gut-brain signaling. *Prevotella* efficiently ferments complex polysaccharides into propionate, which has been associated with improved insulin sensitivity and reduced inflammation.^73^ Lower *Prevotella* abundance may reflect decreased dietary fiber intake or impaired microbial capacity to metabolize plant-based nutrients, both of which can contribute to metabolic dysfunction.^74^ However, its role appears context dependent. *Prevotella copri* has been positively correlated with obesity and fasting glucose in children,^73^ and linked to poor glycemic outcomes and low vegetable intake in adults with type 2 diabetes.^75^ These contradictory findings highlight the complex, diet- and host-dependent nature of *Prevotella*, which may be beneficial in high-fiber diets but detrimental under metabolic stress. Alistipes is generally considered beneficial in obesity contexts, with roles in bile acid metabolism, anti-inflammatory signaling, and tryptophan pathways tied to serotonin and gut-brain communication.^76^ Collectively, the diminished presence of these taxa in obese individuals may impair microbial resilience and compromise essential signaling pathways that protect against both metabolic disease and cognitive decline. A recent meta-analysis of 16 studies also found that *Alistipes* was significantly reduced in the guts of obese individuals.^77^ In contrast, *Bacteroides* was elevated in obese participants, particularly those with MCI. While species within this genus can have beneficial or pathogenic roles, elevated levels have been associated with Western diets rich in fat and animal protein and may contribute to inflammation and reduced SCFA production. The overrepresentation of *Bacteroides* in the obese-MCI group suggests a potential interaction between metabolic and microbial dysregulation in cognitive decline.^78^ Previous studies have linked Bacteroides to MCI and AD: one RCT found an association between *B. thetaiotaomicron* and MCI in older adults,^79^ while another linked *B. fragilis* to neuroinflammation in an AD mouse model.^80^ Conversely, a 2023 study using imaging and metabolomics linked *Bacteroides*-related pathways (e.g., phenylalanine and unsaturated fatty acid metabolism) to improved cognition.^81^ Their research is one of the first to suggest a positive linkage of *Bacteroides* to cognition. Among CN participants, microbial differences were largely attenuated, except for persistent reductions in *Christensenellaceae_R-7_group* among those with obesity. This family is consistently associated with leanness, lower inflammation, and improved metabolic profiles,^82^ and may promote microbiome stability by limiting energy harvest. Notably, lower *Christensenellaceae_R-7_group* abundance has been associated with higher amyloid positivity in AD patients.^82^ Notably, recent research has shown that lower abundance of *Christensenellaceae_R-7 group* is associated with higher odds of amyloid positivity in AD patients,^83^ suggesting a link between this SCFA-producing taxon and neurodegeneration.

### 4.2 Modest weight loss yields cognitive benefits in older adults with obesity: The complexity of body composition changes

Despite both groups losing weight over the 10-week intervention, significant between-group differences in body composition persisted. Obese participants began with greater fat mass, visceral and subcutaneous adiposity, and muscle mass, which were not substantially altered, likely due to the short timeframe. The obese group lost more weight on average (~6 kg vs. ~1.2 kg in non-obese), likely reflecting higher baseline body mass and energy expenditure. However, specific compartments such as fat mass and skeletal muscle did not change significantly in either group, suggesting that weight loss was modest relative to total mass and distributed across tissues. Increases in protein and mineral content among the obese group may reflect relative gains in lean mass or measurement artifacts related to larger body size. Alternatively, poor dietary adherence may have limited measurable changes. Notably, weight loss was not a primary goal of the intervention, which emphasized caloric adequacy within dietary pattern guidelines rather than restriction. Both groups demonstrated similar adherence to Mediterranean components (via MEDAS score), and comparable carbohydrate intake was observed, likely due to gradual titration in the MMKD group. These findings highlight the difficulty of eliciting significant body composition shifts, especially in fat distribution, over short interventions. More intensive protocols, including resistance training or stricter dietary regimens, may be needed to produce meaningful changes in fat and muscle compartments.

### 4.3 Differential effects of diet on microbiome and cognition in obese and non-obese cohorts

Post-intervention, both groups exhibited shifts in gut microbiota composition, with alpha- and beta-diversity measures indicating partial convergence in microbial community structure. This suggests that dietary education interventions can beneficially modulate the microbiome, even in the context of obesity. However, the obese group maintained lower microbial richness (Chao1), implying persistent barriers to microbiome recovery, potentially due to dysbiosis, or long-term dietary patterns. Notably, the intervention differentially influenced the Firmicutes:Bacteroidota (F/B) ratio between groups. Obese participants began with an elevated F/B ratio (a trait often associated with enhanced energy harvest and fat storage) and this ratio increased further (from 2.18 to 3.52) after the intervention. This was primarily due to a decline in Bacteroidota rather than a rise in Firmicutes, suggesting a limited or altered response to dietary fiber, possibly reflecting microbiome rigidity linked to chronic metabolic stress. A meta-analysis from 2022 identified that at the phylum level, obese subjects do have elevated F/B ratios (largely driven by elevated firmicutes) compared to non-obese counterparts,^68^ though a more recent paper revealed that elevated F/B ratio in obese participants was not correlated to increases in body mass.^84^ Thus, F/B ratio may reflect host–microbe adaptations to an obesogenic environment rather than directly causing adiposity. In contrast, the non-obese group experienced a modest reduction in F/B ratio (from 2.94 to 2.75), driven by an increase in Bacteroidota. These taxa are proficient in fermenting dietary fiber into beneficial SCFAs like butyrate, which support gut health and may influence brain function.^85^ The observed enrichment of Bacteroidota in the non-obese group may reflect greater microbiome plasticity and responsiveness to the fiber-rich MD. Together, these findings underscore both the microbiome’s capacity to shift in response to diet and the modifying role of obesity in shaping that response, with potential downstream implications for metabolic regulation and cognitive health.

### 4.4 The obesity paradox

The current findings offer nuanced insights into the so-called “obesity paradox”, the observation that in certain contexts, especially among older adults, higher adiposity may correlate with better health outcomes.^86^ In our study, obesity was associated with lower baseline cognitive performance across several domains. However, over the 10-week dietary education intervention, individuals with obesity showed notable cognitive gains, particularly in episodic memory and executive function (PSMT, DCCS), which were correlated with reductions in body weight and fat mass. This suggests that fat loss, especially visceral fat, known for its inflammatory and metabolic burden, may support cognitive improvement.^87^^,^^88^ Despite lower cognitive scores at baseline and post-intervention (e.g., Flanker, RAVLT), obese participants demonstrated improvements in verbal, associative, and episodic memory, suggesting that cognitive plasticity remains intact. These data demonstrate that cognitive gains remain possible in obesity, especially when modest weight loss helps reduce metabolic load.

In contrast, the non-obese group showed a different pattern, one that aligns with the obesity paradox. The counterintuitive association in adults without obesity, where small increases in body fat correlated with improved memory, is unlikely to reflect a causal benefit of adiposity. Several factors may explain this pattern. Individuals with lower baseline fat may depend more on weight stability for metabolic homeostasis, so minor increases may represent a return toward a physiological set point rather than unhealthy gain. A similar directional pattern has been observed in other populations where low baseline adiposity impairs neurocognitive function. For example, systematic reviews in patients with anorexia nervosa show that cognitive performance improves following weight restoration, suggesting that inadequate fat reserves can compromise cognitive processes and that increases in adiposity may normalize metabolic and neuroendocrine function.^89^ Although our cohort is metabolically healthier, this reinforces the concept that modest fat gain in individuals starting with lower adiposity may support, rather than impair cognitive performance.

Furthermore, the current study's age-stratified results emphasize the complexity of the obesity paradox. Age-stratified analyzes further clarified the complexity of the obesity paradox. Among older adults (73–80 y), gains in skeletal muscle mass were strongly associated with cognitive improvements, a relationship absent in younger participants (58–72 y). This suggests that muscle preservation becomes increasingly critical with age, potentially mitigating sarcopenia and its contribution to cognitive decline. Skeletal muscle may exert neuroprotective effects via myokine signaling (e.g., irisin, BDNF), improved insulin sensitivity, and reduced systemic inflammation.^90-92^ As lean mass diminishes with age, maintaining or increasing skeletal muscle may buffer against both physical and cognitive decline. These findings echo prior research showing that resistance training and muscle-preserving interventions improve cognitive outcomes in older adults with excess adiposity.^93^

Overall, the relationship between body composition and cognition appears context dependent. Excess fat and low muscle mass may each contribute to cognitive risk, while interventions targeting muscle retention and metabolic balance may offer neurocognitive benefits. Future research should further explore the interplay between fat distribution, lean mass, and metabolic health to clarify the mechanisms underlying the obesity paradox in aging populations.

### 4.5 Acetate as a double-edged metabolite in cognitive and metabolic pathways

Acetate, a SCFA, is produced primarily by gut microbiota during the fermentation of dietary fibers, such as those found in plant-based foods, which are a hallmark of the MD. It plays an important role in the gut-brain axis, influencing both metabolic and cognitive functions.^94^ In this study, fecal acetate levels inversely correlated with global cognitive improvement in the obese group, suggesting higher fecal acetate is linked to poorer cognition. Elevated fecal acetate may indicate impaired absorption or utilization, reflecting gut dysfunction or dysbiosis, which can increase inflammation via gut permeability and bacterial metabolites like LPS, thereby impairing cognition.^95^ Excess acetate may also disrupt beneficial SCFA balances, notably reducing protective butyrate, and limit acetate availability for brain functions such as energy metabolism and neuroplasticity mediated by G-protein coupled receptors (GPR41/43).^96^^,^^97^ Reduced receptor activation could impair memory and neuroplasticity. Plasma acetate was associated with weight loss in obese participants, highlighting its role in metabolic shifts during body composition changes. Absorbed from the colon into circulation, acetate influences lipid metabolism, glucose regulation, and inflammation.^96^ In obesity, where chronic inflammation prevails, acetate may exacerbate inflammatory pathways, impairing cognition. Evidence from murine models shows that gut microbiota depletion reduces muscle fiber size and strength, effects reversed by acetate supplementation. Mice lacking acetyl-CoA synthase 2, necessary for acetate utilization, exhibit reduced muscle mass and lifespan, indicating acetate’s role as a metabolic substrate for muscle.^98^ A large cohort study found circulating acetate positively associated with gut microbiome diversity and inversely with visceral fat, suggesting a protective cardio-metabolic role.^99^ Conversely, circulating acetate has been reported to stimulate the parasympathetic nervous system, enhancing insulin and ghrelin secretion, which may increase appetite and promote obesity—contrasting with weight loss observed in our cohort.^100^ These findings underscore acetate’s complex role as a metabolic mediator: fecal acetate likely reflects microbiome fermentation and gut function, whereas plasma acetate reflects systemic metabolic responses influencing body composition. The inverse relationship between fecal acetate and cognition warrants further research into gut-derived metabolites’ effects on brain health. Future studies should clarify whether acetate directly contributes to cognitive decline or serves as a marker of metabolic disturbances. Interventions modulating acetate levels via diet, prebiotics, probiotics, or fecal microbiota transplantation may offer therapeutic potential for metabolic and cognitive dysfunction in obesity.

### 4.6 Fecal and plasma metabolites show divergent links to cognitive function and body composition

Several key metabolites, including lactate, citrate, pyruvate, and propionate, were associated with specific domains of cognitive performance. In the obese group, fecal lactate was inversely associated with weight, and higher lactate levels correlated with less cognitive improvement. As a byproduct of anaerobic metabolism, elevated lactate has been shown to reflect metabolic dysregulation and systemic inflammation, potentially impairing brain function.^101^ This supports the notion that lactate could serve as a marker of poor metabolic health, where high lactate concentrations may reflect the body's struggle to efficiently utilize energy, ultimately affecting brain function. Future research could explore whether reducing lactate levels through targeted metabolic interventions or modulating lactate dehydrogenase activity could improve cognitive outcomes. Plasma citric acid, a central intermediate of the tricarboxylic acid (TCA) cycle, was positively associated with Flanker and FName performance, suggesting that efficient energy metabolism supports cognitive function.^102^ Citric acid fuels adenosine triphosphate (ATP) production, and its link to cognition reinforces the importance of mitochondrial health for tasks requiring sustained attention. Dietary or metabolic interventions that enhance citric acid production may support cognitive performance, especially in those with impaired energy metabolism. Similarly, plasma pyruvate, a key metabolite in aerobic respiration, was positively associated with both Flanker and FName scores. Pyruvate's role as a precursor in ATP generation underscores its importance in supporting executive function and cognitive processing.^103^^,^^104^ Elevated pyruvate levels may reflect better cellular respiration and more efficient energy production, facilitating cognitive tasks that require rapid processing and executive control. Future research could examine how pyruvate metabolism and its associated pathways influence cognitive resilience. Fecal propionate, a SCFA with known anti-inflammatory properties, was linked to better cognitive flexibility (DCCS performance). Propionate promotes GLP-1 and PYY secretion from enteroendocrine cells, potentially contributing to reduced food intake and lower body fat, as observed in this study.^105^ Elevated fecal propionate was associated with lower adiposity in our cohort. It has also shown neuroprotective effects in Parkinson’s models through free fatty acid receptor 3 (FFAR3) signaling on enteric neurons.^106^ Gut-derived propionate can bind to the FFAR3 receptor on enteric neurons, activating a signaling pathway that can influence nerve function and reduce inflammation.^106^ However, propionate has also been linked to adverse outcomes such as polycystic ovarian syndrome (PCOS) and insulin resistance.^107^ These conflicting results suggest that its cognitive effects depend on host metabolism and microbial context. Future studies should distinguish between circulating and fecal propionate and examine whether targeting FFAR3 signaling can improve cognitive outcomes. These metabolite findings also align with emerging evidence supporting a gut–muscle axis that may contribute to the cognitive and body-composition effects observed in our cohort. SCFAs and other microbial metabolites can influence skeletal muscle metabolism by regulating AMP-activated protein kinase (AMPK) signaling and mitochondrial function, thereby affecting muscle quality.^51^ Recent clinical data further support this connection. In the PROMOTe randomized controlled trial, targeted modulation of the gut microbiome led to measurable improvements in both muscle function and cognition in older adults, suggesting that microbial metabolites may exert coordinated effects across muscle and brain systems.^52^

## 5 Conclusions

This pilot study contributes new evidence and insights into how obesity in older adults may shape gut microbiome diversity and cognitive function. These findings reinforce obesity as a metabolic disorder that may heighten predisposition to cognitive decline via alterations in gut microbial ecology and metabolite signaling, a mechanism largely underexplored in aging populations. Few studies have simultaneously examined these domains. Importantly, our study also highlights modifiable pathways that may support cognitive resilience. Participants with obesity showed meaningful memory and executive-function gains following a 10-week dietary education intervention, particularly when reductions in fat mass or increases in skeletal muscle mass occurred. These body-composition–linked cognitive improvements underscore the importance of evaluating adiposity and muscle quality, rather than BMI alone when assessing brain health in aging. The metabolomic data further suggest that gut-derived and circulating metabolites, including acetate, pyruvate, propionate, and amino acids, may represent intermediates linking diet, muscle metabolism, and cognitive performance. Together, the microbial, metabolic, and cognitive results support a growing model of a gut–muscle–brain axis that may help explain individual variability in cognitive aging. As one of the few studies to integrate microbiome, metabolome, cognition, and body composition while stratifying older adults by obesity status, this work addresses a critical gap in the literature. Despite these promising insights, conclusions should be considered exploratory.

This pilot trial has several limitations. With a small sample size (*n* = 31), the analyzes were underpowered to detect modest effects. No power calculations were conducted for this sub-analysis, and while nonparametric statistics were appropriate, they have limited sensitivity. Correlation and regression analyzes were exploratory and should be interpreted with caution. We applied FDR correction to address multiple comparisons in metabolomics, but the study may still be underpowered to detect interaction effects across omics layers. While ANCOVA models adjusted for age and cognitive status, other untested variables such as comorbidities and medications may have influenced outcomes. All body composition–cognition relationships identified here are correlational. Given the small sample size and stratified analyzes, some counterintuitive associations, particularly in the non-obese group, may reflect regression to the mean, measurement variability, or unmeasured confounding rather than true physiological effects. The predominance of female participants (77%) introduces potential sex-related bias, especially given age-related hormonal changes that affect metabolism and muscle mass. Additionally, the 10-week intervention may have been too brief to produce substantial changes in body composition or cognition. The intervention aimed to improve dietary quality and not induce weight loss, via MD or MMKD patterns. Dietary intake was self-reported, introducing potential recall bias, particularly in older adults. Metabolite findings were correlational and limited by uncertainty regarding origin (host vs. microbial), absorption, and bioavailability. Although the NIH Toolbox provides validated cognitive measures, it may not detect subtle or domain-specific changes in older adults or those with MCI. Despite these limitations, this study highlights the potential for lifestyle interventions to improve cognitive and metabolic health in older adults, even those with obesity. Future studies should build on these findings by employing larger cohorts and longer intervention periods that permit evaluation of trajectory, durability, and directionality of changes. Incorporating more sensitive tools such as DEXA for compartment-specific body composition, targeted metabolomics for SCFAs, neurotransmitters and amino acid pathways, and inflammatory biomarkers (e.g., IL-6, CRP, TNF-*α*) will help clarify underlying mechanisms. Interventions that directly target components of the gut–muscle–brain axis—including structured resistance training, prebiotic or microbiome-directed strategies, and integrated nutrition education—should be tested to determine whether modifying these pathways can enhance cognitive resilience in older adults with obesity. Additionally, future work should examine sex-specific responses, the role of visceral vs. subcutaneous fat, and whether baseline microbiome or metabolite profiles can serve as predictors of intervention responsiveness. As the aging population grows, empowering individuals with accessible strategies to support gut health, preserve muscle, and reduce metabolic burden offers a promising approach to enhance cognitive resilience. These findings lay the groundwork for future, larger trials to further investigate gut-brain mechanisms and test the efficacy of non-pharmacologic interventions like nutrition education and lifestyle modification.

## Supplementary Material

Supplementary material Patoine_S1_S5 (1).zip

## Data Availability

The microbiome sequencing data generated and analyzed during this study that support our findings are publicly available in National Center for Biotechnology Information (NCBI)-Sequence Read Archive (SRA) at https://www.ncbi.nlm.nih.gov/sra, reference number PRJNA1272075. Full link: https://www.ncbi.nlm.nih.gov/bioproject/PRJNA1272075. Please reference this ID in any correspondence or future data submissions. Additional data described in the manuscript can be available upon reasonable request to the corresponding author.

## Clinical trial registry number

Identifier NCT06121986, A Scalable Nutrition Adherence Intervention; 2023 Dec. Available from: https://clinicaltrials.gov/ct2/show/NCT06121986.

## Abbreviations

ADRD: Alzheimer’s Disease and Related Dementias
BMI: Body Mass Index
MCI: Mild Cognitive Impairment
AD: Alzheimer’s Disease
NIH: National Institutes of Health
NMR: Nuclear Magnetic Resonance
IL-6: Interleukin-6
TNF- α: Tumor Necrosis Factor-Alpha
IL-1β: Interleukin-1 beta
ROS: Reactive Oxygen Species
LPS: Lipopolysaccharide
MD: Mediterranean Diet
MMKD: Modified Mediterranean Ketogenic Diet
MoCA: Montreal Cognitive Assessment
CN: Cognitively Normal
HIPAA: Health Insurance Portability and Accountability Act.
MI: Motivational Interviewing
CBT: Cognitive Behavioral Therapy
BIA: Bioelectrical Impedance Analysis
DCCS: Dimensional Change Card Sort Test
FName: Face Name Associative Memory Exam Delay
Flanker: Flanker Inhibitory Control and Attention Task
LSWM: List Sorting Working Memory Test
PSMT: Picture Sequence Memory Test
RAVLT: Rey Auditory Verbal Learning Test
RAVLD: Rey Auditory Verbal Learning Delay
QIIME2: Quantitative Insights into Microbial Ecology
ASV: Amplicon Sequence Variants
TSP: Trimethylsilyl Propionate
FDR: False Discovery Rate
ANCOVA: Analysis of Covariance
MEDAS: Mediterranean Diet Adherence Screener
SCFA: Short-chain Fatty Acid
TMA: Trimethylamine
BDNF: Brain-derived Neurotrophic factor
TCA: Tricarboxylic Acid
ATP: Adenosine Triphosphate
FFAR3: Free Fatty Acid Receptor 3
PCOS: Polycystic Ovarian Syndrome
AMPK: AMP-Activated Protein Kinase
DEXA: Dual-energy X-ray Absorptiometry

## References

[1] Fakhouri TH. Prevalence of obesity among older adults in the United States. 2012. pp. 2007–2010 US Department of Health and Human Services, Centers for Disease Control.

[2] Emmerich SD, Fryar CD, Stierman B, Ogden CL. Obesity and severe obesity prevalence in adults: United States, August 2021–August 2023. 2024 10.15620/cdc/159281 PMC11744423 39808758

[3] Chandrasekaran P, Weiskirchen R. The role of obesity in type 2 diabetes mellitus—an overview. Int J Mol Sci. 2024;25:1882. doi: 10.3390/ijms25031882. 38339160 PMC10855901

[4] Welsh A, Hammad M, Piña IL, Kulinski J. Obesity and cardiovascular health. Eur J Prev Cardiol. 2024;31:1026–1035. doi: 10.1093/eurjpc/zwae025. 38243826 PMC11144464

[5] Neeland IJ, Lim S, Tchernof A, Gastaldelli A, Rangaswami J, Ndumele CE, Powell-Wiley TM, Després J-P. Metabolic syndrome. Nat Rev Dis Primer. 2024;10:77. doi: 10.1038/s41572-024-00563-5. 39420195

[6] Gudala K, Bansal D, Schifano F, Bhansali A. Diabetes mellitus and risk of dementia: a meta‐analysis of prospective observational studies. J Diabetes Investig. 2013;4:640–650. doi: 10.1111/jdi.12087. PMC4020261 24843720

[7] Wei J, Zhu X, Liu J, Gao Y, Liu X, Wang K, Zheng X. Estimating global prevalence of mild cognitive impairment and dementia in elderly with overweight, obesity, and central obesity: a systematic review and meta‐analysis. Obes Rev. 2025;26:e13882. doi: 10.1111/obr.13882. 39647849

[8] Picone P, Di Carlo M, Nuzzo D. Obesity and Alzheimer’s disease: Molecular bases. Eur J Neurosci. 2020;52:3944–3950. doi: 10.1111/ejn.14758. 32323378

[9] Guillemot-Legris O, Muccioli GG. Obesity-induced neuroinflammation: beyond the hypothalamus. Trends Neurosci. 2017;40:237–253. doi: 10.1016/j.tins.2017.02.005. 28318543

[10] Alexaki VI. The impact of obesity on microglial function: Immune, metabolic and endocrine perspectives. Cells. 2021;10:1584. doi: 10.3390/cells10071584. 34201844 PMC8307603

[11] Selman A, Burns S, Reddy AP, Culberson J, Reddy PH. The role of obesity and diabetes in dementia. Int J Mol Sci. 2022;23:9267. doi: 10.3390/ijms23169267. 36012526 PMC9408882

[12] Wong Zhang DE, Tran V, Vinh A, Dinh QN, Drummond GR, Sobey CG, Jelinic M, De Silva TM. Pathophysiological links between obesity and dementia. NeuroMolecular Med. 2023;25:451–456. doi: 10.1007/s12017-023-08746-1. 37086380 PMC10721659

[13] Varra F-N, Varras M, Varra V-K. Theodosis-Nobelos P. Molecular and pathophysiological relationship between obesity and chronic inflammation in the manifestation of metabolic dysfunctions and their inflammation‑mediating treatment options. Mol Med Rep. 2024;29:95. doi: 10.3892/mmr.2024.13219. 38606791 PMC11025031

[14] Ungvari Z, Toth P, Tarantini S, Prodan CI, Sorond F, Merkely B, Csiszar A. Hypertension-induced cognitive impairment: from pathophysiology to public health. Nat Rev Nephrol. 2021;17:639–654. doi: 10.1038/s41581-021-00430-6. 34127835 PMC8202227

[15] Goodpaster BH, Park SW, Harris TB, Kritchevsky SB, Nevitt M, Schwartz AV, Simonsick EM, Tylavsky FA, Visser M, Newman AB. The loss of skeletal muscle strength, mass, and quality in older adults: the health, aging and body composition study. J Gerontol A Biol Sci Med Sci. 2006;61:1059–1064. doi: 10.1093/gerona/61.10.1059. 17077199

[16] Ling Z, Liu X, Cheng Y, Yan X, Wu S. Gut microbiota and aging. Crit Rev Food Sci Nutr. 2022;62:3509–3534. doi: 10.1080/10408398.2020.1867054. 33377391

[17] Prado CM, Batsis JA, Donini LM, Gonzalez MC, Siervo M. Sarcopenic obesity in older adults: a clinical overview. Nat Rev Endocrinol. 2024;20:261–277. doi: 10.1038/s41574-023-00943-z. 38321142 PMC12854800

[18] Saito H, Matsue Y, Kamiya K, Kagiyama N, Maeda D, Endo Y, Ueno H, Yoshioka K, Mizukami A, Saito K. Sarcopenic obesity is associated with impaired physical function and mortality in older patients with heart failure: insight from FRAGILE-HF. BMC Geriatr. 2022;22:556. doi: 10.1186/s12877-022-03168-3. 35787667 PMC9254413

[19] Ishii S, Chang C, Tanaka T, Kuroda A, Tsuji T, Akishita M, Iijima K. The association between sarcopenic obesity and depressive symptoms in older Japanese adults. PLoS One. 2016;11:e0162898. doi: 10.1371/journal.pone.0162898. 27627756 PMC5023182

[20] Atmis V, Yalcin A, Silay K, Ulutas S, Bahsi R, Turgut T, Mut Sürmeli D, Selvi Öztorun H, Yaman S, Çoşarderelioğlu Ç. The relationship between all-cause mortality sarcopenia and sarcopenic obesity among hospitalized older people. Aging Clin Exp Res. 2019;31:1563–1572. doi: 10.1007/s40520-019-01277-5. 31350700

[21] Weng X, Liu S, Li M, Zhang Y, Zhang Y, Liu C, Zhu J, Hu H. Relationship between sarcopenic obesity and cognitive function in patients with mild to moderate Alzheimer’s disease. Psychogeriatrics. 2023;23:944–953. doi: 10.1111/psyg.13015. 37652079

[22] Palacios S, Orozco R, Garcia-Almeida JM. Beyond body mass index: redefining the diagnosis of obesity. Gynecol Endocrinol. 2025;41:2480332. doi: 10.1080/09513590.2025.2480332. 40111135

[23] Sanford AM. Mild cognitive impairment. Clin Geriatr Med. 2017;33:325–337. doi: 10.1016/j.cger.2017.02.005. 28689566

[24] Kivipelto M, Mangialasche F, Ngandu T. Lifestyle interventions to prevent cognitive impairment, dementia and Alzheimer disease. Nat Rev Neurol. 2018;14:653–666. doi: 10.1038/s41582-018-0070-3. 30291317

[25] Siervo M, Shannon OM, Llewellyn DJ, Stephan BC, Fontana L. Mediterranean diet and cognitive function: From methodology to mechanisms of action. Free Radic Biol Med. 2021;176:105–117. doi: 10.1016/j.freeradbiomed.2021.09.018. 34562607

[26] Valls-Pedret C, Sala-Vila A, Serra-Mir M, Corella D, de la Torre R, Martínez-González MÁ, Martínez-Lapiscina EH, Fitó M, Pérez-Heras A, Salas-Salvadó J. Mediterranean diet and age-related cognitive decline: a randomized clinical trial. JAMA Intern Med. 2015;175:1094–1103. doi: 10.1001/jamainternmed.2015.1668. 25961184

[27] Muscogiuri G, Verde L, Sulu C, Katsiki N, Hassapidou M, Frias-Toral E, Cucalón G, Pazderska A, Yumuk VD, Colao A. Mediterranean diet and obesity-related disorders: what is the evidence?. Curr Obes Rep. 2022;11:287–304. doi: 10.1007/s13679-022-00481-1. 36178601 PMC9729142

[28] Barber TM, Kabisch S, Pfeiffer AF, Weickert MO. The effects of the mediterranean diet on health and gut microbiota. Nutrients. 2023;15:2150. doi: 10.3390/nu15092150. 37432307 PMC10180651

[29] Ghosh TS, Rampelli S, Jeffery IB, Santoro A, Neto M, Capri M, Giampieri E, Jennings A, Candela M, Turroni S, et al. Mediterranean diet intervention alters the gut microbiome in older people reducing frailty and improving health status: the NU-AGE 1-year dietary intervention across five European countries. Gut. 2020;69:1218–1228. doi: 10.1136/gutjnl-2019-319654. 32066625 PMC7306987

[30] Mazza E, Ferro Y, Pujia R, Mare R, Maurotti S, Montalcini T, Pujia A. Mediterranean diet in healthy aging. J Nutr Health Aging. 2021;25:1076–1083. doi: 10.1007/s12603-021-1675-6. 34725664 PMC8442641

[31] García-Casares N, Gallego Fuentes P, Barbancho MÁ, López-Gigosos R, García-Rodríguez A, Gutiérrez-Bedmar M. Alzheimer’s disease, mild cognitive impairment and Mediterranean diet. A systematic review and dose-response meta-analysis. J Clin Med. 2021;10:4642. doi: 10.3390/jcm10204642. 34682764 PMC8537524

[32] Aburto MR, Cryan JF. Gastrointestinal and brain barriers: unlocking gates of communication across the microbiota–gut–brain axis. Nat Rev Gastroenterol Hepatol. 2024;21:222–247. doi: 10.1038/s41575-023-00890-0. 38355758

[33] Schneider E, O’Riordan KJ, Clarke G, Cryan JF. Feeding gut microbes to nourish the brain: unravelling the diet–microbiota–gut–brain axis. Nat Metab. 2024;6:1454–1478. doi: 10.1038/s42255-024-01108-6. 39174768

[34] Liu S, Gao J, Zhu M, Liu K, Zhang H-L. Gut microbiota and dysbiosis in Alzheimer’s disease: implications for pathogenesis and treatment. Mol Neurobiol. 2020;57:5026–5043. doi: 10.1007/s12035-020-02073-3. 32829453 PMC7541367

[35] Amabebe E, Robert FO, Agbalalah T, Orubu ES. Microbial dysbiosis-induced obesity: role of gut microbiota in homoeostasis of energy metabolism. Br J Nutr. 2020;123:1127–1137. doi: 10.1017/S0007114520000380. 32008579

[36] Dao MC, Everard A, Aron-Wisnewsky J, Sokolovska N, Prifti E, Verger EO, Kayser BD, Levenez F, Chilloux J, Hoyles L. Akkermansia muciniphila and improved metabolic health during a dietary intervention in obesity: relationship with gut microbiome richness and ecology. Gut. 2016;65:426–436. doi: 10.1136/gutjnl-2014-308778. 26100928

[37] Tavella T, Rampelli S, Guidarelli G, Bazzocchi A, Gasperini C, Pujos-Guillot E, Comte B, Barone M, Biagi E, Candela M. Elevated gut microbiome abundance of Christensenellaceae, Porphyromonadaceae and Rikenellaceae is associated with reduced visceral adipose tissue and healthier metabolic profile in Italian elderly. Gut Microbes. 2021;13:1880221. doi: 10.1080/19490976.2021.1880221. 33557667 PMC7889099

[38] Wang L, He X, Zhang Z, Chen N. Distinct Gut Microbiota Signatures in Order People with Sarcopenia without Obesity and Sarcopenic Obesity. 2024 10.1016/j.clnu.2025.04.004 40252601

[39] Forst AA, Heston MB, González A, Chin NA, Przybelski RJ, Johnson SC, Asthana S, Knight R, Kaddurah‐Daouk R, Rey FE. Bacteroides genus is associated with lower executive function in cognitively unimpaired participants. Alzheimers Dement. 2022;18:e069291. doi: 10.1002/alz.069291.

[40] Dong TS, Guan M, Mayer EA, Stains J, Liu C, Vora P, Jacobs JP, Lagishetty V, Chang L, Barry RL. Obesity is associated with a distinct brain-gut microbiome signature that connects Prevotella and Bacteroides to the brain’s reward center. Gut Microbes. 2022;14:2051999. doi: 10.1080/19490976.2022.2051999. 35311453 PMC8942409

[41] Schoultz I, Claesson MJ, Dominguez‐Bello MG, Fåk Hållenius F, Konturek P, Korpela K, Laursen MF, Penders J, Roager H, Vatanen T. Gut microbiota development across the lifespan: Disease links and health‐promoting interventions. J Intern Med. 2025;297:560–583. doi: 10.1111/joim.20089. 40270478 PMC12087861

[42] Golshany H, Helmy SA, Morsy NFS, Kamal A, Yu Q, Fan L. The gut microbiome across the lifespan: How diet modulates our microbial ecosystem from infancy to the elderly. Int J Food Sci Nutr. 2025;76:95–121. doi: 10.1080/09637486.2024.2437472. 39701663

[43] Lim MY, Nam Y-D. Gut microbiome in healthy aging versus those associated with frailty. Gut Microbes. 2023;15:2278225. doi: 10.1080/19490976.2023.2278225. 37968837 PMC10730223

[44] Bradley E, Haran J. The human gut microbiome and aging. Gut Microbes. 2024;16:2359677. doi: 10.1080/19490976.2024.2359677. 38831607 PMC11152108

[45] Agus A, Clément K, Sokol H. Gut microbiota-derived metabolites as central regulators in metabolic disorders. Gut. 2021;70:1174–1182. doi: 10.1136/gutjnl-2020-323071. 33272977 PMC8108286

[46] Perry RJ, Peng L, Barry NA, Cline GW, Zhang D, Cardone RL, Petersen KF, Kibbey RG, Goodman AL, Shulman GI. Acetate mediates a microbiome–brain–β-cell axis to promote metabolic syndrome. Nature. 2016;534:213–217. doi: 10.1038/nature18309. 27279214 PMC4922538

[47] Wu L, Han Y, Zheng Z, Peng G, Liu P, Yue S, Zhu S, Chen J, Lv H, Shao L. Altered gut microbial metabolites in amnestic mild cognitive impairment and Alzheimer’s disease: signals in host–microbe interplay. Nutrients. 2021;13:228. doi: 10.3390/nu13010228. 33466861 PMC7829997

[48] Connell E, Le Gall G, Pontifex MG, Sami S, Cryan JF, Clarke G, Müller M, Vauzour D. Microbial-derived metabolites as a risk factor of age-related cognitive decline and dementia. Mol Neurodegener. 2022;17:43. doi: 10.1186/s13024-022-00548-6. 35715821 PMC9204954

[49] Meslier V, Laiola M, Roager HM, De Filippis F, Roume H, Quinquis B, Giacco R, Mennella I, Ferracane R, Pons N. Mediterranean diet intervention in overweight and obese subjects lowers plasma cholesterol and causes changes in the gut microbiome and metabolome independently of energy intake. Gut. 2020;69:1258–1268. doi: 10.1136/gutjnl-2019-320438. 32075887 PMC7306983

[50] Grosicki GJ, Fielding RA, Lustgarten MS. Gut microbiota contribute to age-related changes in skeletal muscle size, composition, and function: biological basis for a gut-muscle axis. Calcif Tissue Int. 2018;102:433–442. doi: 10.1007/s00223-017-0345-5. 29058056 PMC5858871

[51] Prokopidis K, Chambers E, Ni Lochlainn M, Witard OC. Mechanisms linking the gut-muscle axis with muscle protein metabolism and anabolic resistance: implications for older adults at risk of sarcopenia. Front Physiol. 2021;12:770455. doi: 10.3389/fphys.2021.770455. 34764887 PMC8576575

[52] Ni Lochlainn M, Bowyer RC, Moll JM, García MP, Wadge S, Baleanu A-F, Nessa A, Sheedy A, Akdag G, Hart D. Effect of gut microbiome modulation on muscle function and cognition: the PROMOTe randomised controlled trial. Nat Commun. 2024;15:1859. doi: 10.1038/s41467-024-46116-y. 38424099 PMC10904794

[53] Hochuli N, Kadyan S, Park G, Patoine C, Nagpal R. A Gut Microbial Metabolite Alleviates Stress-Induced Neurobehavioral Dysfunction in an Alzheimer’s Disease Model. Mol Neurobiol. 2025;62:1–15. doi: 10.1007/s12035-025-04960-z. 40310548

[54] Park G, Kadyan S, Hochuli N, Salazar G, Laitano O, Chakrabarty P, Efron PA, Zafar MA, Wilber A, Nagpal R. An Enteric Bacterial Infection Triggers Neuroinflammation and Neurobehavioral Impairment in 3xTg-AD transgenic mice. J Infect Dis. 2024;230:S95–108. doi: 10.1093/infdis/jiae165. 39255397 PMC11385593

[55] Munley JA, Park G, Kelly LS, Kannan KB, Mankowski RT, Casadesus G, Chakrabarty P, Wallet SM, Maile R, Bible LE. Persistence and sexual dimorphism of gut dysbiosis and pathobiome after sepsis and trauma. Ann Surg. 2024;280:491–503. doi: 10.1097/SLA.0000000000006385. 38864230 PMC11392637

[56] Park G, Kadyan S, Hochuli N, Pollak J, Wang B, Salazar G, Chakrabarty P, Efron P, Sheffler J, Nagpal R. A modified Mediterranean-style diet enhances brain function via specific gut-microbiome-brain mechanisms. Gut Microbes. 2024;16:2323752. doi: 10.1080/19490976.2024.2323752. 38444392 PMC10936641

[57] Park G, Johnson K, Miller K, Kadyan S, Singar S, Patoine C, Hao F, Lee Y, Patterson AD, Arjmandi B. Almond snacking modulates gut microbiome and metabolome in association with improved cardiometabolic and inflammatory markers. Npj Sci Food. 2025;9:35. doi: 10.1038/s41538-025-00403-0. 40113782 PMC11926229

[58] Kadyan S, Park G, Wang B, Nagpal R. Dietary fiber modulates gut microbiome and metabolome in a host sex-specific manner in a murine model of aging. Front Mol Biosci. 2023;10:1182643. doi: 10.3389/fmolb.2023.1182643. 37457834 PMC10345844

[59] Callahan B, McMurdie P, Rosen M, Han A, Johnson A, Dada SH. DADA2: High-resolution sample inference from Illumina amplicon data. Nat Methods. 2016;13:581–583. 27214047 10.1038/nmeth.3869 PMC4927377

[60] Katoh K, Misawa K, Kuma K, Miyata T. MAFFT: a novel method for rapid multiple sequence alignment based on fast Fourier transform. Nucleic Acids Res. 2002;30:3059–3066. 12136088 10.1093/nar/gkf436 PMC135756

[61] Quast C, Pruesse E, Yilmaz P, Gerken J, Schweer T, Yarza P, Peplies J, Glöckner FO. The SILVA ribosomal RNA gene database project: improved data processing and web-based tools. Nucleic Acids Res. 2012;41:D590–6. doi: 10.1093/nar/gks1219. 23193283 PMC3531112

[62] Kadyan S, Park G, Wang B, Singh P, Arjmandi B, Nagpal R. Resistant starches from dietary pulses modulate the gut metabolome in association with microbiome in a humanized murine model of ageing. Sci Rep. 2023;13:10566. doi: 10.1038/s41598-023-37036-w. 37386089 PMC10310774

[63] Gratton J, Phetcharaburanin J, Mullish BH, Williams HR, Thursz M, Nicholson JK, Holmes E, Marchesi JR, Li JV. Optimized sample handling strategy for metabolic profiling of human feces. Anal Chem. 2016;88:4661–4668. doi: 10.1021/acs.analchem.5b04159. 27065191

[64] Wang B, Maldonado-Devincci AM, Jiang L. Evaluating line-broadening factors on a reference spectrum as a bucketing method for NMR based metabolomics. Anal Biochem. 2020;606:113872. doi: 10.1016/j.ab.2020.113872. 32738215 PMC7484314

[65] Torres-Fuentes C, Schellekens H, Dinan TG, Cryan JF. The microbiota–gut–brain axis in obesity. Lancet Gastroenterol Hepatol. 2017;2:747–756. doi: 10.1016/S2468-1253(17)30147-4. 28844808

[66] Balasubramanian P, Kiss T, Tarantini S, Nyúl-Tóth Á, Ahire C, Yabluchanskiy A, Csipo T, Lipecz A, Tabak A, Institoris A. Obesity-induced cognitive impairment in older adults: a microvascular perspective. Am J Physiol-Heart Circ Physiol. 2021;320:H740–61. doi: 10.1152/ajpheart.00736.2020. 33337961 PMC8091942

[67] Jung C-H, Mok J-O. Recent updates on associations among various obesity metrics and cognitive impairment: from body mass index to sarcopenic obesity. J Obes Metab Syndr. 2022;31:287–295. doi: 10.7570/jomes22058. 36530066 PMC9828704

[68] Pinart M, Dötsch A, Schlicht K, Laudes M, Bouwman J, Forslund SK, Pischon T, Nimptsch K. Gut microbiome composition in obese and non-obese persons: a systematic review and meta-analysis. Nutrients. 2021;14:12. doi: 10.3390/nu14010012. 35010887 PMC8746372

[69] Gong J, Shen Y, Zhang H, Cao M, Guo M, He J, Zhang B, Xiao C. Gut microbiota characteristics of people with obesity by meta-analysis of existing datasets. Nutrients. 2022;14:2993. doi: 10.3390/nu14142993. 35889949 PMC9325184

[70] Abuqwider JN, Mauriello G, Altamimi M. Akkermansia muciniphila, a new generation of beneficial microbiota in modulating obesity: a systematic review. Microorganisms. 2021;9:1098. doi: 10.3390/microorganisms9051098. 34065217 PMC8161007

[71] Zhang Y, Liu R, Chen Y, Cao Z, Liu C, Bao R, Wang Y, Huang S, Pan S, Qin L. Akkermansia muciniphila supplementation in patients with overweight/obese type 2 diabetes: Efficacy depends on its baseline levels in the gut. Cell Metab. 2025;37:592–605.e6. doi: 10.1016/j.cmet.2024.12.010. 39879980

[72] Panzetta ME, Valdivia RH. Akkermansia in the gastrointestinal tract as a modifier of human health. Gut Microbes. 2024;16:2406379. doi: 10.1080/19490976.2024.2406379. 39305271 PMC11418289

[73] Ley RE. Prevotella in the gut: choose carefully. Nat Rev Gastroenterol Hepatol. 2016;13:69–70. doi: 10.1038/nrgastro.2016.4. 26828918

[74] Gong J, Zhang Q, Hu R, Yang X, Fang C, Yao L, Lv J, Wang L, Shi M, Zhang W. Effects of Prevotella copri on insulin, gut microbiota and bile acids. Gut Microbes. 2024;16:2340487. doi: 10.1080/19490976.2024.2340487. 38626129 PMC11028016

[75] Tsai C-Y, Liu P-Y, Huang M-C, Chang C-I, Chen H-Y, Chou Y-H, Tsai C-N, Lin C-H. Abundance of Prevotella copri in gut microbiota is inversely related to a healthy diet in patients with type 2 diabetes. J Food Drug Anal. 2023;31:599. 38526814 10.38212/2224-6614.3484 PMC10962673

[76] Parker BJ, Wearsch PA, Veloo AC, Rodriguez-Palacios A. The genus Alistipes: gut bacteria with emerging implications to inflammation, cancer, and mental health. Front Immunol. 2020;11:906. doi: 10.3389/fimmu.2020.00906. 32582143 PMC7296073

[77] Chanda D, De D. Meta-analysis reveals obesity associated gut microbial alteration patterns and reproducible contributors of functional shift. Gut Microbes. 2024;16:2304900. doi: 10.1080/19490976.2024.2304900. 38265338 PMC10810176

[78] Wang C, Zhao J, Zhang H, Lee Y-K, Zhai Q, Chen W. Roles of intestinal bacteroides in human health and diseases. Crit Rev Food Sci Nutr. 2021;61:3518–3536. doi: 10.1080/10408398.2020.1802695. 32757948

[79] Aljumaah MR, Bhatia U, Roach J, Gunstad J, Peril MAA. The gut microbiome, mild cognitive impairment, and probiotics: a randomized clinical trial in middle-aged and older adults. Clin Nutr. 2022;41:2565–2576. doi: 10.1016/j.clnu.2022.09.012. 36228569

[80] Xia Y, Xiao Y, Wang Z-H, Liu X, Alam AM, Haran JP, McCormick BA, Shu X, Wang X, Ye K. Bacteroides Fragilis in the gut microbiomes of Alzheimer’s disease activates microglia and triggers pathogenesis in neuronal C/EBPβ transgenic mice. Nat Commun. 2023;14:5471. doi: 10.1038/s41467-023-41283-w. 37673907 PMC10482867

[81] Zhang S, Qian Y, Li Q, Xu X, Li X, Wang C, Cai H, Zhu J, Yu Y. Metabolic and neural mechanisms underlying the associations between gut bacteroides and cognition: a large-scale functional network connectivity study. Front Neurosci. 2021;15:750704. doi: 10.3389/fnins.2021.750704. 34733135 PMC8558260

[82] Waters JL, Ley RE. The human gut bacteria Christensenellaceae are widespread, heritable, and associated with health. BMC Biol. 2019;17:1–11. 31660948 10.1186/s12915-019-0699-4 PMC6819567

[83] Verhaar BJ, Hendriksen HM, de Leeuw FA, Doorduijn AS, van Leeuwenstijn M, Teunissen CE, Barkhof F, Scheltens P, Kraaij R, van Duijn CM. Gut microbiota composition is related to AD pathology. Front Immunol. 2022;12:794519. doi: 10.3389/fimmu.2021.794519. 35173707 PMC8843078

[84] Karačić A, Renko I, Krznarić Ž, Klobučar S, Liberati Pršo A-M. The Association between the Firmicutes/Bacteroidetes Ratio and Body Mass among European Population with the Highest Proportion of Adults with Obesity: An Observational Follow-Up Study from Croatia. Biomedicines. 2024;12:2263. doi: 10.3390/biomedicines12102263. 39457576 PMC11505267

[85] Magne F, Gotteland M, Gauthier L, Zazueta A, Pesoa S, Navarrete P, Balamurugan R. The firmicutes/bacteroidetes ratio: a relevant marker of gut dysbiosis in obese patients?. Nutrients. 2020;12:1474. doi: 10.3390/nu12051474. 32438689 PMC7285218

[86] Antonopoulos AS, Tousoulis D. The molecular mechanisms of obesity paradox. Cardiovasc Res. 2017;113:1074–1086. doi: 10.1093/cvr/cvx106. 28549096

[87] Hunter GR, Gower BA, Kane BL. Age related shift in visceral fat. Int J Body Compos Res. 2010;8:103. 24834015 PMC4018766

[88] Yoon DH, Choi SH, Yu JH, Ha JH, Ryu SH, Park DH. The relationship between visceral adiposity and cognitive performance in older adults. Age Ageing. 2012;41:456–461. doi: 10.1093/ageing/afs018. 22440588

[89] Hemmingsen SD, Wesselhoeft R, Lichtenstein MB, Sjögren JM, Støving RK. Cognitive improvement following weight gain in patients with anorexia nervosa: a systematic review. Eur Eat Disord Rev. 2021;29:402–426. doi: 10.1002/erv.2796. 33044043

[90] Oudbier SJ, Goh J, Looijaard SMLM, Reijnierse EM, Meskers CGM, Maier AB. Pathophysiological mechanisms explaining the association between low skeletal muscle mass and cognitive function. J Gerontol Ser A. 2022;77:1959–1968. doi: 10.1093/gerona/glac121. PMC9536455 35661882

[91] Liu C, Wong PY, Chow SKH, Cheung WH, Wong RMY. Does the regulation of skeletal muscle influence cognitive function? A scoping review of pre-clinical evidence. J Orthop Transl. 2023;38:76–83. doi: 10.1016/j.jot.2022.10.001. PMC9619139 36381246

[92] Bilski J, Pierzchalski P, Szczepanik M, Bonior J, Zoladz JA. Multifactorial mechanism of sarcopenia and sarcopenic obesity. Role of physical exercise, microbiota and myokines. Cells. 2022;11:160. doi: 10.3390/cells11010160. 35011721 PMC8750433

[93] Hsu K-J, Liao C-D, Tsai M-W, Chen C-N. Effects of exercise and nutritional intervention on body composition, metabolic health, and physical performance in adults with sarcopenic obesity: a meta-analysis. Nutrients. 2019;11:2163. doi: 10.3390/nu11092163. 31505890 PMC6770949

[94] Mann ER, Lam YK, Uhlig HH. Short-chain fatty acids: linking diet, the microbiome and immunity. Nat Rev Immunol. 2024;24:577–595. doi: 10.1038/s41577-024-01014-8. 38565643

[95] Jung H, Lee D, You H, Lee M, Kim H, Cheong E, Um JW. LPS induces microglial activation and GABAergic synaptic deficits in the hippocampus accompanied by prolonged cognitive impairment. Sci Rep. 2023;13:6547. doi: 10.1038/s41598-023-32798-9. 37085584 PMC10121592

[96] Silva Y, Bernardi A, Frozza R. The role of short-chain fatty acids from gut microbiota in gut-brain communication. Front Endocrinol (Lausanne). 2020;11:25. doi: 10.3389/fendo.2020.00025. 32082260 PMC7005631

[97] Xu M, Jiang Z, Wang C, Li N, Bo L, Zha Y, Bian J, Zhang Y, Deng X. Acetate attenuates inflammasome activation through GPR43-mediated Ca2+-dependent NLRP3 ubiquitination. Exp Mol Med. 2019;51:1–13. 10.1038/s12276-019-0276-5 PMC6802670 31337751

[98] Kobayashi S, Morino K, Okamoto T, Tanaka M, Ida S, Ohashi N, Murata K, Yanagimachi T, Sakai J, Maegawa H. Acetate derived from the intestinal tract has a critical role in maintaining skeletal muscle mass and strength in mice. Physiol Rep. 2024;12:e16047. doi: 10.14814/phy2.16047. 38837588 PMC11150057

[99] Nogal A, Louca P, Zhang X, Wells PM, Steves CJ, Spector TD, Falchi M, Valdes AM, Menni C. Circulating levels of the short-chain fatty acid acetate mediate the effect of the gut microbiome on visceral fat. Front Microbiol. 2021;12:711359. doi: 10.3389/fmicb.2021.711359. 34335546 PMC8320334

[100] Perry RJ, Peng L, Barry NA, Cline GW, Zhang D, Cardone RL, Petersen KF, Kibbey RG, Goodman AL, Shulman GI. Acetate mediates a microbiome–brain–β-cell axis to promote metabolic syndrome. Nature. 2016;534:213–217. doi: 10.1038/nature18309. 27279214 PMC4922538

[101] Li X, Yang Y, Zhang B, Lin X, Fu X, An Y, Zou Y, Wang J-X, Wang Z, Yu T. Lactate metabolism in human health and disease. Signal Transduct Target Ther. 2022;7:305. doi: 10.1038/s41392-022-01151-3. 36050306 PMC9434547

[102] Książek E. Citric acid: properties, microbial production, and applications in industries. Molecules. 2023;29:22. doi: 10.3390/molecules29010022. 38202605 PMC10779990

[103] Gonzalez SV, Nguyen NH, Rise F, Hassel B. Brain metabolism of exogenous pyruvate. J Neurochem. 2005;95:284–293. doi: 10.1111/j.1471-4159.2005.03365.x. 16181432

[104] Yako H, Niimi N, Kato A, Takaku S, Tatsumi Y, Nishito Y, Kato K, Sango K. Role of pyruvate in maintaining cell viability and energy production under high-glucose conditions. Sci Rep. 2021;11:18910. doi: 10.1038/s41598-021-98082-w. 34556698 PMC8460646

[105] Chambers ES, Viardot A, Psichas A, Morrison DJ, Murphy KG, Zac-Varghese SE, MacDougall K, Preston T, Tedford C, Finlayson GS. Effects of targeted delivery of propionate to the human colon on appetite regulation, body weight maintenance and adiposity in overweight adults. Gut. 2015;64:1744–1754. doi: 10.1136/gutjnl-2014-307913. 25500202 PMC4680171

[106] Hou Y, Shan C, Zhuang S, Zhuang Q, Ghosh A, Zhu K, Kong X, Wang S, Gong Y, Yang Y. Gut microbiota-derived propionate mediates the neuroprotective effect of osteocalcin in a mouse model of Parkinson’s disease. Microbiome. 2021;9:1–17. doi: 10.1186/s40168-020-00988-6. 33517890 PMC7849090

[107] Dong S, Yao X, Jiao J, Lin B, Yan F, Wang X. Fecal propionate is a signature of insulin resistance in polycystic ovary syndrome. Front Cell Infect Microbiol. 2025;14:1394873. doi: 10.3389/fcimb.2024.1394873. 39872943 PMC11769941

