# Supplementary figures and images for: Obesity-associated gut microbiome influences diet-induced metabolic and cognitive outcomes in older adults
Source: Gut Microbes Rep. 2025 Dec 25;3(1):2605879. doi: 10.1080/29933935.2025.2605879 (PMC12938881; doi:10.1080/29933935.2025.2605879)

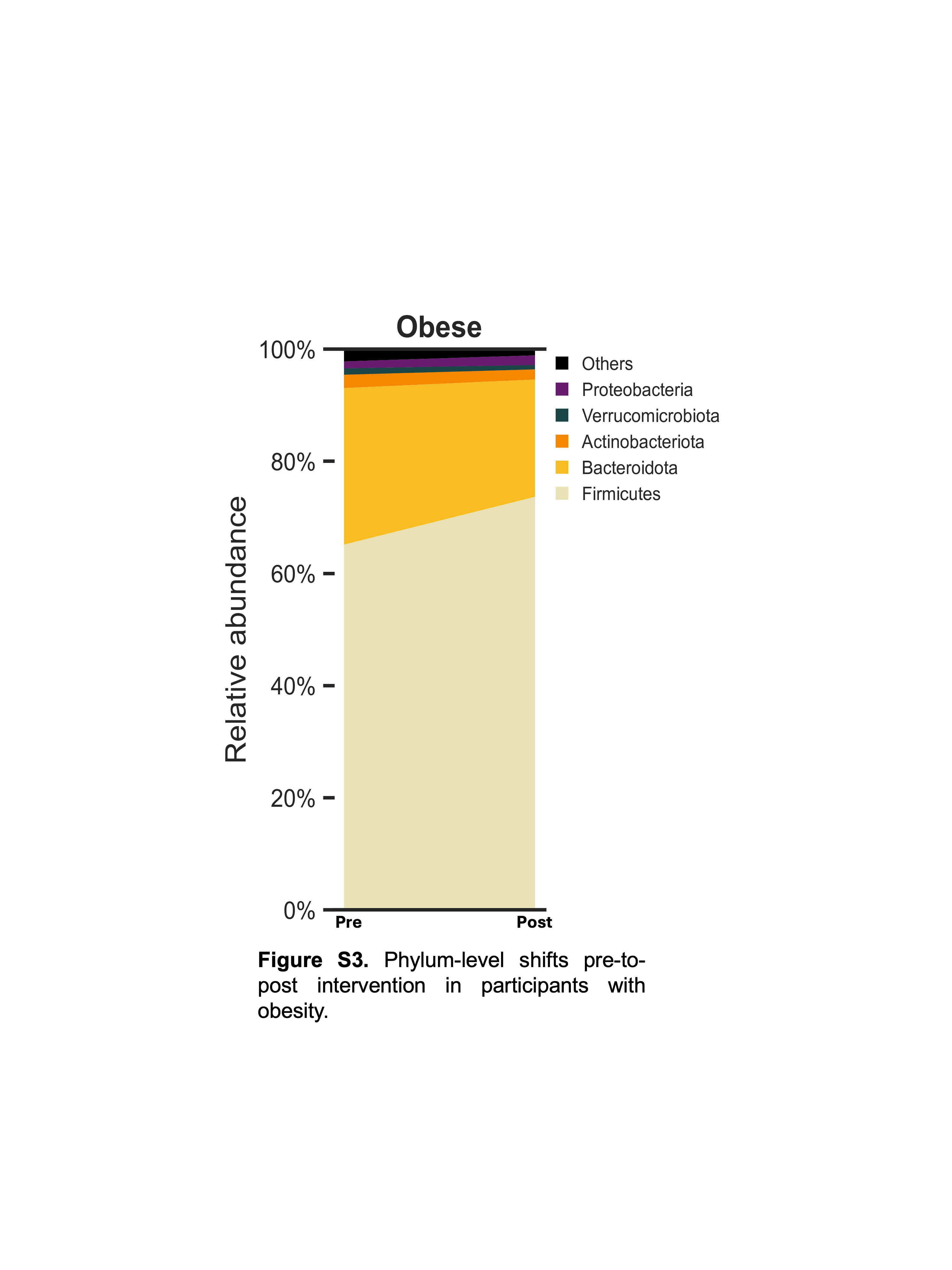

Supplement: Supplementary material — Patoine_S1_S5 (1).zip [file KGMR_A_2605879_SM9595.zip › Patoine_S1_S5/Figure S3.tiff]

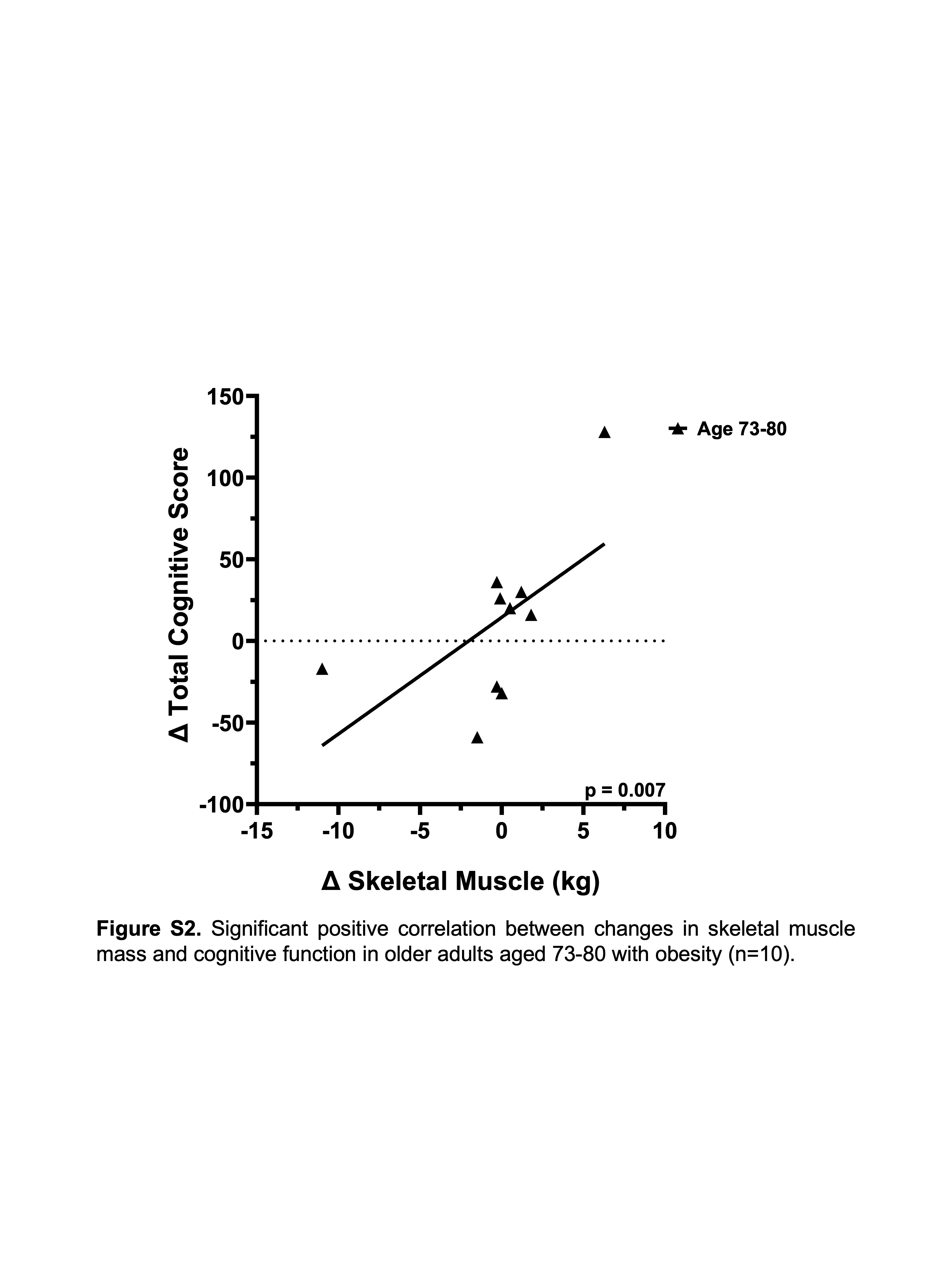

Supplement: Supplementary material — Patoine_S1_S5 (1).zip [file KGMR_A_2605879_SM9595.zip › Patoine_S1_S5/Figure S2.tiff]

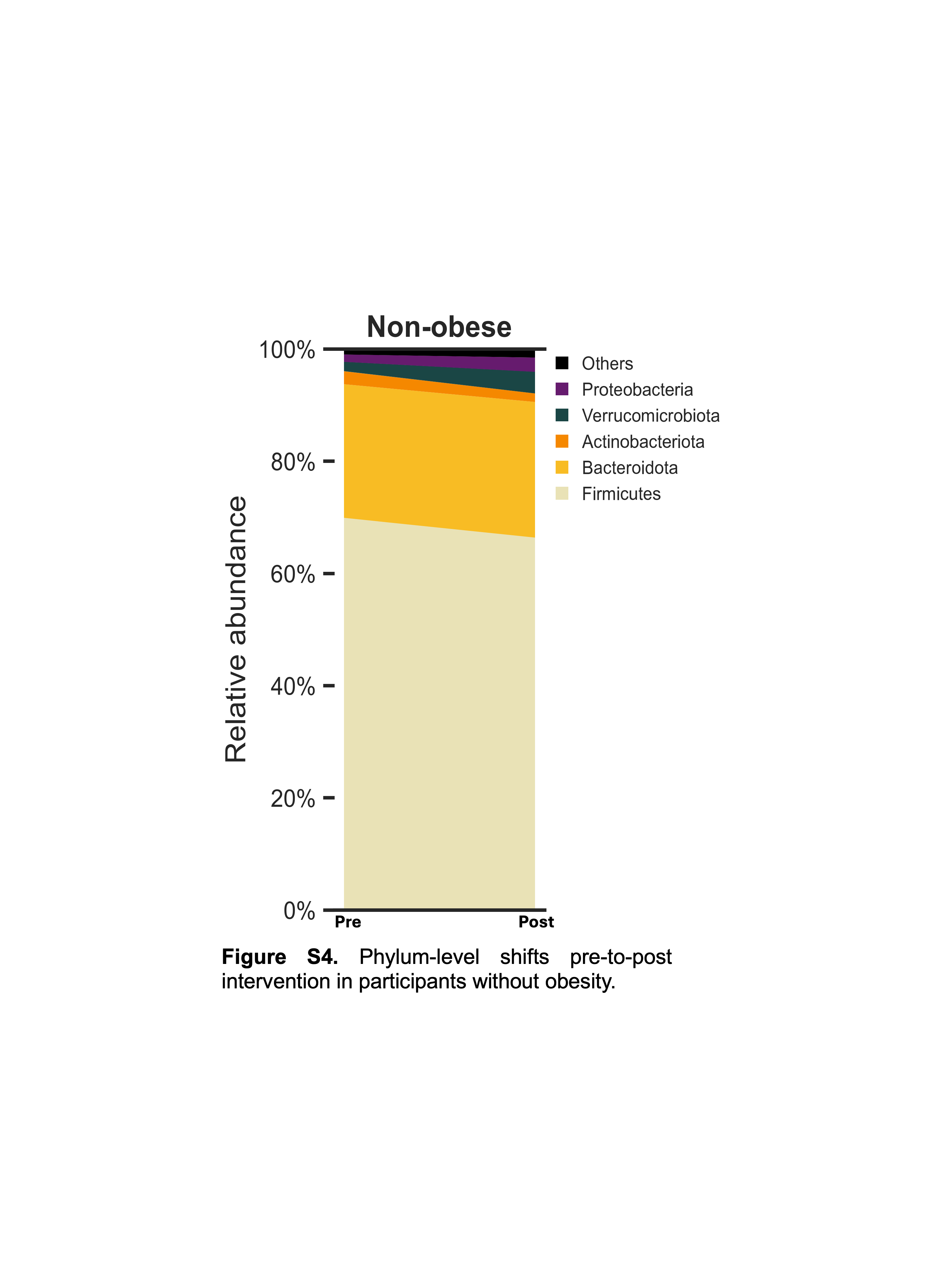

Supplement: Supplementary material — Patoine_S1_S5 (1).zip [file KGMR_A_2605879_SM9595.zip › Patoine_S1_S5/Figure S4..tiff]

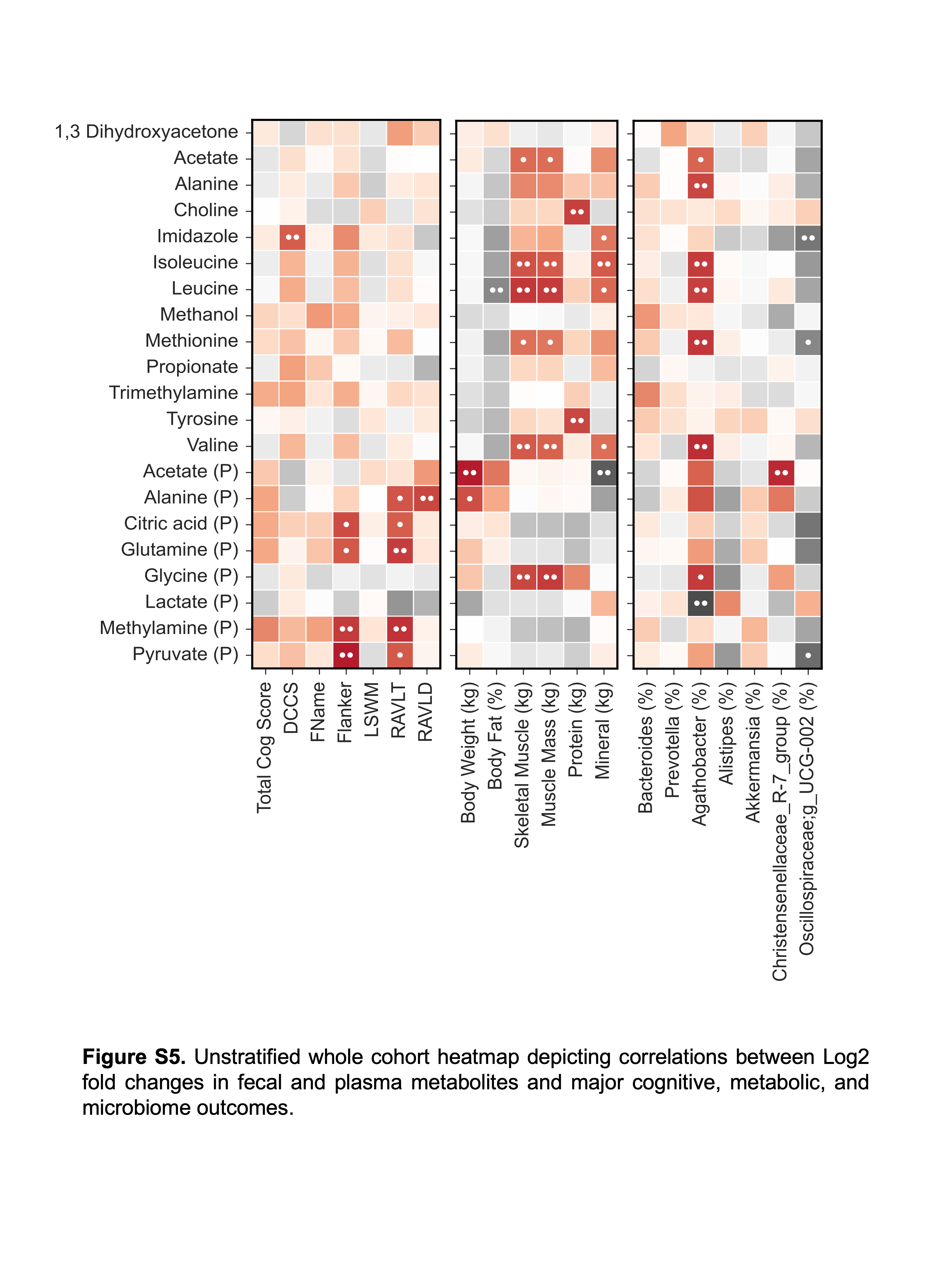

Supplement: Supplementary material — Patoine_S1_S5 (1).zip [file KGMR_A_2605879_SM9595.zip › Patoine_S1_S5/Figure S5.tiff]

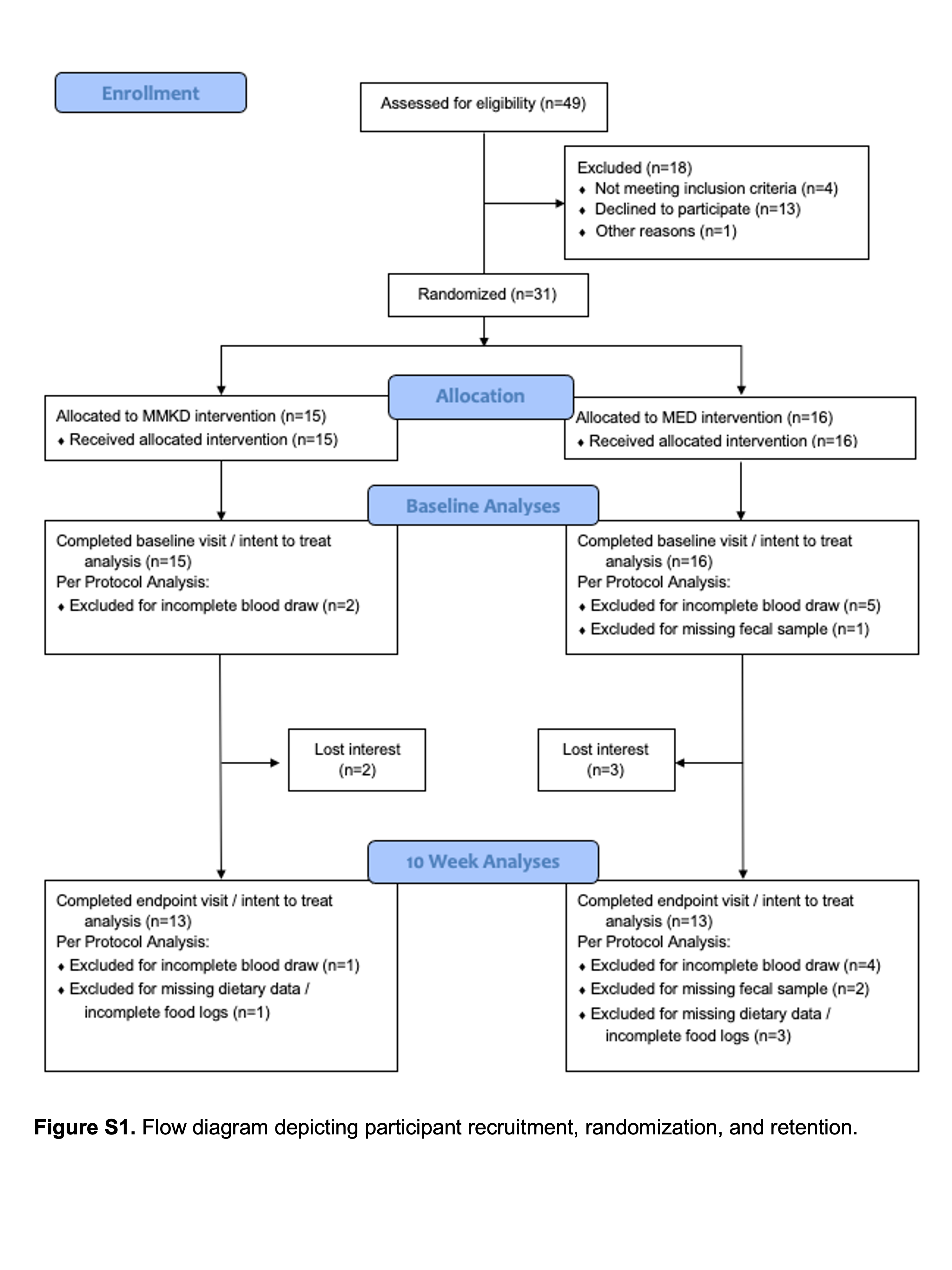

Supplement: Supplementary material — Patoine_S1_S5 (1).zip [file KGMR_A_2605879_SM9595.zip › Patoine_S1_S5/Figure S1.tiff]
